# Supplementary material for: Circulating Exosomal miRNA Profiles Predict the Occurrence and Recurrence of Hepatocellular Carcinoma in Patients with Direct-Acting Antiviral-Induced Sustained Viral Response
Source: Biomedicines. 2019 Nov 3;7(4):87. doi: 10.3390/biomedicines7040087 (PMC6966514; doi:10.3390/biomedicines7040087)
Supplement: Supplementary file 1 [file biomedicines-07-00087-s001.pdf]

Circulating Exosomal miRNA Profiles Predict the Occurrence and Recurrence of Hepatocellular Carcinoma in Patients with Direct-Acting Antiviral-Induced Sustained Viral Response

Saori Itami-Matsumoto, Michiyo Hayakawa, Sawako Uchida-Kobayashi, Masaru Enomoto, Akihiro Tamori, Kazuyuki Mizuno, Hidenori Toyoda, Takeyuki Tamura, Tatsuya Akutsu, Takahiro Ochiya, Norifumi Kawada and Yoshiki Murakami

Table S1. Clinical background in detail.

			pre treatment							
**Case No**	**HCC**	**SVR**	**ALT (IU/l)**	**T.Bil (mg/dl)**	**Alb (g/dl)**	**PT (%)**	**PLT (x10⁴/mm)**	**AFP (ng/ml)**	**M2BPGi**	**FIB-4**
AD-2		2015/2/25	131	1.2	3.5	62	10.3	62.1	7.48	7.87
AD-3		2015/3/2	30	1.9	3.1	NT	9.9	49	6.13	7.81
AD-5		2015/3/5	58	0.8	3.7	NT	6.6	7.3	6.93	8.93
AD-6		2015/3/9	78	0.7	3.5	84	6.7	60	5.98	9.01
AD-11		2015/3/25	59	0.8	4	76	8.9	56.8	7.07	5.01
AD-12		2015/4/1	101	0.6	3.2	71	9.1	375.7	14.18	8.77
AD-27		2015/4/27	33	0.9	3.6	81	8.3	4.6	4.47	6.46
AD-33		2015/4/28	49	0.8	3.3	99	8.4	3.4	3.51	7.96
AD-36		2015/5/5	93	0.5	4.6	103	3.7	5.8	1.75	
AD-38		2015/4/28	39	1.8	3.7	72	11.1	10.2	5.22	4.14
AD-39		2015/5/19	50	1.2	NT	92	5.6	17.1	11.09	14.32
AD-51		2015/5/6	28	1.2	3.3	78	14.2	26.2	6.89	2.85
AD-52		2015/5/12	32	1	2.8	95	14.7	9.9	8.19	7.29
AD-56		2015/2/16	51	0.5	3.2	64	6.5	32.2	6.23	9.33
AD-58		2015/1/5	41	1.9	3.9	85	3.9	2.9	9.38	11.25
AD-70		2015/7/6	27	0.5	4.3	106	16	3.5	3.23	3.33
AD-82		2015/7/2	105	0.6	3.7	85	9.5	5.4	2.77	6.73
AD-84		2015/7/9	149	0.8	4.5	NT	14.9	4	2.04	3.11
AD-94		2015/7/19	40	0.8	3.1	90	16.2	48.6	9.37	3.97
AD-96		2015/6/17	85	0.7	3.5	97	9.3	39	4.8	4.69
AD-97		2015/7/20	68	0.4	3.5	70	6	20.7	10.21	11.27
AD-104		2015/8/5	104	0.6	4	130	9.7	8.1	2.56	5.20

AD-105	2015/8/3	42	0.9	3.2	63	4.2	100.9	8.85	17.82
AD-113	2015/9/21	32	0.7	3.8	NT	19.5	11.7	5.26	2.68
AD-122	2015/9/16	72	0.9	3.9	79	5.9	70.3	7.68	11.07
AD-123	2015/9/27	97	1	4.5	82	13.8	14.6	2.26	2.87
AD-129	2015/9/21	35	1	3.5	90	6.9	54.2	10.4	6.38
AD-131	2015/9/20	54	0.6	3.3	102	17.2	6.3	4.56	3.19
AD-132	2015/9/21	39	0.4	3.2	109	11	6.8	3.57	5.26
AD-137	2015/9/30	62	1.2	3.1	72	10.2	9.3	11.16	7.00
AD-142	2015/10/26	99	0.5	3.4	70	11.3	13.8	3.06	4.87
AD-154	2016/2/19	36	0.5	3.3	108	9.5	6.5	9.3	5.68
SL-27	2015/12/29	43	0.8	3.6	89	6.4	31.2	8.83	10.37
SL-49	2016/1/20	47	0.6	3.4	74	9.2	4.2	6.54	6.00
SL-65	2016/2/5	54	0.6	3.7	86	7.7	6	4.23	8.19
SL-108	2016/3/19	54	0.9	3	68	15.7	3.2	2.95	2.34
SL-162	2016/5/12	28	1	3.3	54	10.8	2.7	2.79	5.35
SL-208	2016/6/27	62	0.9	3.9	74	7.4	8.4	3.42	5.51
SL-99	2016/3/10	34	1.4	3.3	66	4.2	2.3	9.74	2.48
SL-220	2016/7/9	41	1	3.8	81	29.5	6.5	4.21	2.10
SL-226	2016/7/15	34	1.7	3.7	48	7.8	16.7	7.92	10.33
SL-268	2016/8/26	47	1.2	3.4	75	5.2	179.8	10.8	11.15
SL-284	2016/9/11	27	1.8	4.3	82	4.9	5.1	6.88	12.82
SR-8	2015/10/3	27	0.9	3.1	54	6.4	17.1	5.92	9.88
SR-59	2015/11/23	57	1.9	3.4	78	5	7.6	4.83	16.82
SR-81	2015/12/15	29	0.5	3.9	84	10.8	23.9	11.45	5.43
SL-154	2016/5/4	149	0.7	3.5	77	13.7	213.7	9.42	5.09
SL-155	2016/5/5	45	0.7	4	92	14.9	5.1	1.65	2.82
SL-157	2016/5/7	21	0.6	3.6	92	18.4	NT	1.85	1.93
SL-159	2016/5/9	101	1.2	3.7	56	10.1	9.2	10.95	6.43
SL-160	2016/5/10	38	0.8	4.1	104	13.8	7.9	1.21	2.74
SL-163	2016/5/13	17	0.5	4.4	100	23.9	4.8	1.26	1.12
SL-165	2016/5/15	34	0.8	3.9	82	15.3	7	1.31	3.81
SL-166	2016/5/16	96	0.7	3.8	66	15.8	135	4.58	3.78

SL-168		2016/5/18	34	0.5	4.3	89	25.7	2.7	3.61	1.06
AD-1	2015/3/25	2015/2/19	38	0.6	4.1	NT	21.7	18	2.44	1.86
AD-7	2017/4/24	2015/3/13	19	0.6	4.1	77	7.6	4.2	3.79	4.65
AD-26	2015/12/25	2015/4/16	31	0.4	2.8	90	4.4	71.7	8.18	10.83
AD-59	2016/4/4	2015/5/19	28	1.5	3.8	79	8.4	6.8	3.25	7.82
AD-116	2017/5/12	2015/8/25	46	0.9	2.9	88	6.5	34.2	5.17	13.50
SL-35	2016/8/24	2016/1/6	80	0.6	3.8	86	5.4	8	5.76	8.73
SL-44	2016/8/12	2016/1/15	62	0.4	4.4	81	8	5.1	1.62	6.02
SL-48	2016/8/30	2016/1/19	43	0.4	4.3	105	17.8	9.4	4.83	3.63
SL-153	2017/4/25	2016/5/3	58	0.5	3.5	88	22	6.4	2.51	2.81
SL-173	2016/5/23	2016/5/23	33	0.5	3.4	72	20.6	6.8	4.6	2.88
SL-192	2016/12/19	2016/6/11	86	0.5	3.3	75	15	42.4	6.58	5.41
SL-293	2016/12/8	2016/9/20	39	0.5	4	29	15.3	34.1	4.09	3.56
SL-341	2017/6/22	2016/11/7	100	1.1	4	91	11.6	17	1.67	4.53
SR-3	2016/4/22	2015/9/28	63	0.8	3.7	81	9.5	11.6	3	2.75
SR-16	2016/6/30	2015/10/11	91	1.2	4.5	95	20.1	2.9	0.8	2.66
AD-10		2015/4/1	29	0.6	3.6	75	8.5	6.3	3.25	5.57
AD-40		2015/4/29	30	0.9	4.1	101	8.8	5.8	1.11	5.10
AD-44		2015/5/5	73	1	3.8	84	10.8	24	4.31	7.80
AD-45		2015/1/6	39	0.9	3.8	78	11.7	19.4	8.88	3.94
AD-49		2015/4/27	24	0.5	3.6	58	24.2	3.8	1.51	2.54
AD-50		2015/5/3	46	0.7	3.7	92	20.3	5.5	1.47	2.55
AD-53		2015/5/12	29	1.1	3.4	79	12.1	3.2	3.01	4.37
AD-67		2015/6/8	113	0.7	3.4	88	13.4	27.2	7.79	5.60
AD-92		2015/8/3	65	0.8	3.4	83	9.2	6.4	3.74	8.82
AD-109		2015/3/18	50	1.2	3.8	90	8	19.2	7.33	7.12
AD-111		2015/8/14	9	0.9	3.5	79	11.7	3.5	0.83	4.25
AD-117		2015/8/31	28	0.6	3.7	82	12.4	3.7	1.24	3.14
AD-120		2015/9/6	24	0.5	3.1	109	18.8	<2.0	3.1	2.99
AD-136		2015/9/28	45	2	3.1	78	5.2	295	12.42	9.84
AD-150		2015/11/30	44	0.8	4	97	17.2	7.5	1.72	3.46
SL-2		2015/12/4	42	1.2	3.9	79	10.5	89.1	15.8	5.12
SL-6		2015/12/8	32	0.6	3.6	91	6.2	3.3	5.56	9.75

SL-20		2015/12/22	54	0.6	4.4	90	13.2	13.9	1.54	3.68
SL-40		2016/1/11	42	0.5	3.2	79	8.9	6.4	7.91	9.45
SL-52		2016/1/23	113	0.6	3.7	65	13.1	8.9	1.39	3.54
SL-82		2016/2/22	23	0.5	4	89	11.21	4.3	1.08	6.96
SL-87		2016/2/27	104	0.4	3.4	89	6.3	23.6	8.28	12.24
SL-90		2016/3/1	64	1	3.6	83	8.3	46	9.13	6.79
SL-92		2016/3/3	55	0.6	3.6	94	15.9	21	3.14	3.15
SL-101		2016/3/12	166	0.6	4.1	92	17.2	17.6	3.3	3.80
SL-106		2016/3/17	16	0.6	3.9	132	16.9	4.6	1.93	3.32
SL-117		2016/3/28	37	0.6	3.3	90	14	13.2	12.94	4.16
SL-132		2016/4/12	19	0.5	3.9	66	9.8	2.8	2.94	5.12
SL-152		2016/5/2	22	0.6	4.3	76	20.6	2.7	1.55	2.33
SL-161		2016/5/11	21	0.4	4.2	101	24.9	4.2	1.63	1.89
SL-198		2016/6/17	32	0.6	3.8	76	15.8	17.9	4.29	5.02
SL-205		2016/6/24	15	0.8	3.4	70	11.3	8.7	8.92	5.60
SL-224		2016/7/13	22	0.7	3.9	100	16	2.4	1.56	2.37
SL-228		2016/7/17	53	0.8	4.5	75	8.6	10.1	4	8.35
SL-234		2016/7/23	30	0.4	3.9	84	28.3	<2.0	2	1.81
SL-257		2016/8/15	34	0.6	3.7	79	9.3	19	3.92	5.81
SL-263		2016/8/21	35	0.8	3.4	78	10.5	5.4	4.58	7.48
SL-272		2016/8/30	35	0.6	3.2	75	6	10.7	7.36	9.79
SL-294		2016/9/21	34	0.4	3.7	101	28.3	4.7	1.81	1.91
SL-301		2016/9/28	42	0.8	3.8	107	21.6	3.6	1.7	1.93
SL-323		2016/10/20	118	0.7	3.5	98	2.6	8.8	NT	
SR-53		2015/11/17	61	1.3	4	93	7.1	5.3	0.99	7.01
SR-88		2015/12/22	82	0.4	3.8	103	16	12.5	5.91	3.24
SR-168		2016/3/11	109	1	3.6	86	8.2	23.3	7.45	8.50
AD-22	2015/4/22	2015/3/31	124	0.7	NT	79	7.7	57.1	6.79	6.80
AD-24	2015/7/9	2015/4/16	22	1.4	3.5	62	6.6	3.2	3.85	6.56
AD-47	2015/7/2	2015/5/5	51	0.6	3.2	79	6.6	7.9	7.54	7.23
AD-54	2016/12/21	2015/5/13	21	0.3	3.3	129	7.1	8.7	3.59	7.75
AD-57	2015/6/11	2015/5/18	22	0.4	3.1	78	15.1	8.3	2.22	2.76
AD-75	2015/11/26	2015/6/25	21	0.9	3.6	82	7.7	<2.0	1.99	5.03

AD-106	2015/11/25	2015/8/4	66	0.5	3.8	111	20.1	10.5	4.19	2.95
AD-138	2016/7/12	2015/10/5	15	0.7	2.9	80	7.7	25.1	14.54	12.65
AD-148	2016/7/23	2015/11/11	83	0.5	3.3	87	8.8	20.5	10.31	7.79
AD-151	2016/12/1	2015/11/30	22	0.3	3.8	103	11.5	6.8	4.5	3.52
SL-15	2016/4/7	2015/12/17	141	0.7	3.7	91	12.2	224.4	10.37	6.79
SL-46	2016/7/8	2016/1/17	39	1	2.9	66	5.8	236.1	7.96	10.23
SL-121	2016/8/8	2016/4/1	53	0.8	3.9	82	11.1	4.3	2.33	5.21
SL-137	2016/5/10	2016/4/17	75	0.7	4	90	14.1	7.5	1.8	3.24
SL-151	2016/7/8	2016/5/1	56	0.9	2.4	62	7.8	7.2	3.56	7.09
SL-158	2017/5/19	2016/5/8	109	0.9	3.7	74	8.7	213.4	8.96	8.16
SL-167	2017/6/26	2016/5/17	22	0.4	3.7	84	22	3.5	2.76	2.53
SL-248	2017/6/19	2016/8/6	85	1.1	3.9	86	10.3	6.5	2.32	7.33
SL-252		2016/8/10	71	1.2	3.5	82	4.1	26	12.1	19.46
SR-6	2017/2/15	2015/10/1	41	0.8	3.1	23	7.7	34.7	14.66	13.99
SR-14	2016/9/7	2015/10/9	46	0.7	3.2	88	16.9	8.7	3.69	3.39
SR-23	2016/3/23	2015/10/18	14	0.5	4.5	93	16.6	3.9	0.9	2.74
SR-80	2016/12/13	2015/12/14	67	0.9	3.5	75	10.7	9.2	7.95	8.50
SR-103	2016/9/23	2016/1/6	11	1.2	4.4	84	14.6	5.5	1.43	4.92
SR-158	2017/6/12	2016/3/1	59	0.6	3.1	61	7.5	12	8.23	8.86

A

	Pre Treatment						Post Treatment			
**case No**	**clinical feature**	**age**	**sex**	**ALT**	**T.Bil**	**Alb**	**PT**	**PLT**	**AFP**	**M2BPGi**
AD-2	LC	64	M	40	0.7	3.8	69	9.6	9.9	2.62
AD-3	LC	77	F	14	0.8	3.7	95	8.9	10.3	2.26
AD-5	LC	67	F	72	0.4	3.8	74	8.5	5	4.74
AD-6	LC	65	F	16	0.8	4.4	NT	8.4	8.2	1.5
AD-11	LC	59	M	19	1.1	4.2	71	10.3	8.5	2.2
AD-12	LC	48	F	50	0.7	3.6	66	9.9	25.9	6.83
AD-27	LC	79	F	10	0.8	3.7	76	8.2	2.3	4.01
AD-33	LC	72	F	31	0.9	3.6	84	7.8	2.8	NT
AD-36	LC	53	M	35	0.4	4.6	99	3.9	3.4	NT
AD-38	LC	70	F	15	1.4	3.8	74	10.4	3.6	NT
AD-39	LC	63	M	14	0.9	4.1	93	6.5	4.1	5.01
AD-51	LC	51	M	26	1.1	3.6	79	12.4	15.7	NT
AD-52	LC	73	F	44	1.7	4.3	NT	10.3	5.5	4.27
AD-56	LC	71	M	24	0.5	2.5	64	17	4.9	2.99
AD-58	LC	53	M	20	1.6	2.9	57	2.6	NT	9.74
AD-70	LC	77	F	11	0.3	4.4	108	15.5	2.4	NT
AD-82	LC	78	F	NT	0.7	4	74	8.2	4.7	2.39
AD-84	LC	59	M	46	0.9	4.4	90	15.4	2.6	0.58
AD-94	LC	74	F	10	0.7	3.6	81	15	12.4	3.64
AD-96	LC	49	M	26	0.4	4.3	116	10.8	10.9	6.96
AD-97	LC	82	F	46	0.7	4	63	7.9	5.3	2.16

AD-104	LC	66	F	26	0.5	4.1	103	13	NT	NT
AD-105	LC	77	F	23	0.7	4.3	76	6.1	8.4	5.37
AD-113	LC	72	F	23	0.7	3.7	72	18.8	6.9	NT
AD-122	LC	77	M	NT	0.8	4.4	NT	6.1	7.8	1.87
AD-123	LC	63	F	34	0.8	4.4	75	12.9	6.5	0.78
AD-129	LC	62	M	9	1	41	97	8.3	10.8	2.62
AD-131	LC	65	F	12	0.7	3.8	103	19.4	2.6	NT
AD-132	LC	86	M	19	0.6	3.7	108	9.6	4.3	2.39
AD-137	LC	76	F	15	1.2	3.9	78	11.7	3.6	NT
AD-142	LC	76	F	54	1.2	3.5	61	9.9	13.1	NT
AD-154	LC	60	M	22	0.3	2.8	NT	16.1	6.5	NT
SL-27	LC	50	M	27	0.7	3.9	85	7.6	11.6	3.7
SL-49	LC	62	F	23	0.7	3.5	70	11.1	4	3.59
SL-65	LC	76	F	28	0.8	3.7	71	8.3	4.9	2.82
SL-108	LC	54	M	15	0.8	3	68	16.7	2.1	NT
SL-162	LC	68	M	18	1.4	3.7	68	11.5	2.9	1.52
SL-208	LC	73	F	19	0.9	4	81	8.4	6.9	2.19
SL-99	LC	54	M	27	1.8	3.6	69	5.6	2.4	4.78
SL-220	LC	64	F	40	2	4.2	91	28.3	6.4	2.92
SL-226	LC	77	F	27	2.3	3.2	52	6.7	9.6	5.73
SL-268	LC	75	F	17	1	3.8	85	7.5	23.9	4.58
SL-284	LC	48	M	23	1.2	3.8	81	4.1	4.1	3.61
SR-8	LC	73	F	27	0.7	3.8	68	7.8	10.6	4.98
SR-59	LC	69	F	18	2.8	3.5	75	5.9	5.5	5.14
SR-81	LC	77	M	27	0.4	4.1	82	12.9	19.4	6.29
SL-154	CH	51	M	67	0.8	3.9	75	14.5	22	4.61
SL-155	CH	67	M	24	0.8	4.1	102	14.6	3.7	0.81
SL-157	CH	58	M	18	0.5	3.9	104	24.3	2.2	1.09
SL-159	LC	51	M	181	0.8	4	60	13.1	7.2	3.4
SL-160	CH	53	M	17	1	4.2	100	17.6	6.2	0.67
SL-163	CH	65	M	15	0.5	3.9	95	20.9	2.8	0.67
SL-165	CH	83	F	14	0.6	3.8	91	12.8	6.9	0.68
SL-166	CH	76	M	73	0.7	3.7	68	14.4	29.5	3.47
SL-168	CH	51	F	24	0.6	3.8	94	31.4	<2.0	2.09
AD-1	CH	69	F	16	0.9	4.5	99	18.7	190.5	NT
AD-7	LC	67	F	16	0.7	4.2	NT	5.5	3.9	1.88
AD-26	LC	68	F	18	0.8	4	104	5.1	NT	5.95
AD-59	LC	74	F	17	1.7	4	81	10.3	6.5	3.34
AD-116	LC	85	M	12	0.8	3.8	87	9.5	2.9	2.5
SL-35	CH	68	F	18	0.5	4.2	84	7	10.4	NT
SL-44	LC	79	M	30	0.6	4.2	81	9.1	3.8	0.97
SL-48	CH	80	M	24	0.6	4.6	91	23.1	6.8	NT
SL-153	CH	76	F	25	0.6	3.7	92	20.9	5.6	1.31
SL-173	CH	74	F	58	0.6	2.6	67	16.6	3.7	2.99
SL-192	CH	74	M	16	0.7	3.8	88	17.4	12.8	1.5
SL-293	CH	81	F	15	0.4	3.6	NT	17.3	64.3	1.71
SL-341	CH	59	M	33	1.2	4.1	123	16.7	17.6	NT
SR-3	CH	56	M	17	0.9	3.9	75	13.6	4.2	1.55
SR-16	CH	67	M	35	1.1	4.5	79	22	NT	NT
AD-10	LC	75	F	23	1	4.3	86	10.3	NT	3.59
AD-40	CH	82	M	32	0.7	4.3	85	8.6	2.8	0.65
AD-44	LC	72	M	15	0.8	4.2	87	12.2	15.9	2.43
AD-45	LC	64	F	551	2.2	3.8	53	18.8	8.2	4.68
AD-49	CH	67	M	12	0.8	3.9	82	26.9	NT	1.04
AD-50	CH	73	M	30	0.4	3.6	84	18.5	3.7	1.73
AD-53	LC	77	M	14	1.1	3.6	73	10.5	2	2.76

AD-67	CH	84	M	13	0.5	3.8	87	13.4	6.7	1.96
AD-92	LC	77	M	17	1.3	3.8	80	8.3	2.4	2.92
AD-109	LC	66	F	30	1.1	3.5	92	7.3	NT	6.89
AD-111	CH	71	M	69	1.1	3.1	65	12.1	15.8	2.05
AD-117	LC	71	F	17	0.6	4	NT	13.9	2.9	0.84
AD-120	CH	81	F	15	0.6	3.4	106	16.5	<2.0	3.06
AD-136	LC	52	F	18	1	4.1	89	5.8	8.1	7.21
AD-150	CH	79	F	11	0.6	4.1	80	16.3	3	0.76
SL-2	LC	72	F	35	1.1	3.8	77	8.8	17.4	9.05
SL-6	LC	76	F	16	0.6	3.2	94	8	2.7	5.83
SL-20	LC	70	M	29	0.9	4.3	74	14.2	9.8	0.83
SL-40	LC	79	F	11	0.8	3.4	79	8.5	8.1	3.11
SL-52	CH	53	M	13	0.7	4.5	72	12.2	3.9	0.95
SL-82	CH	72	F	15	0.6	3.6	82	10	2.8	1.22
SL-87	LC	71	M	66	0.6	3.8	89	9	11.8	6.4
SL-90	LC	82	M	49	1.2	3.9	69	10.2	23.4	NT
SL-92	LC	79	M	17	0.5	3.3	85	19.9	30	NT
SL-101	CH	68	F	114	1.2	4.2	87	17.1	14.1	2
SL-106	CH	68	M	9	0.7	3.9	107	19.9	4.1	1.13
SL-117	LC	77	F	14	0.7	3.8	78	14.1	5.6	NT
SL-132	LC	81	M	17	0.7	3.7	58	11.8	2.9	1.96
SL-152	CH	75	M	25	0.4	3.9	87	20.4	2	0.96
SL-161	CH	72	M	17	0.7	4.2	107	22.1	4.1	1.1
SL-198	CH	88	F	17	0.6	4.2	96	13.8	8.3	1.27
SL-205	LC	79	F	30	1.2	4.1	69	10.7	8.4	5.93
SL-224	LC	74	M	7	0.7	3.3	98	15.4	2.1	NT
SL-228	LC	83	F	22	1.2	4.4	82	8.8	4.7	2.31
SL-234	CH	78	M	13	0.7	4.4	101	23.1	<2.0	1.14
SL-257	LC	67	F	39	0.8	3.9	93	8.5	6.5	2.31
SL-263	LC	83	F	19	1.1	3.7	81	9.9	6.1	NT
SL-272	LC	79	M	29	0.7	3.2	82	8.5	7.6	3.42
SL-294	CH	79	M	17	0.3	3.7	132	34.7	4.8	0.93
SL-301	CH	75	M	41	0.8	3.7	110	31.9	2.8	1.09
SL-323	CH	57	F	24	0.9	4.1	100	4.7	6.1	2.49
SR-53	LC	67	M	15	1.5	3.7	79	8	4.1	0.68
SR-88	CH	69	F	32	0.4	3.7	92	19.2	7	2.66
SR-168	LC	68	M	14	1.1	4	83	12.4	8.1	1.99
AD-22	LC	67	M	59	0.5	4.4	71	9.4	45.1	1.93
AD-24	LC	70	M	12	0.9	3.8	70	7.6	3.1	2.49
AD-47	LC	71	F	24	0.7	4.1	81	5.7	6.3	5.33
AD-54	CH	72	F	20	0.8	3.9	110	6.1	5.9	2.72
AD-57	CH	63	M	21	0.4	4	79	16.3	3.4	1.66
AD-75	LC	74	M	13	0.9	4.3	90	6.8	<2.0	0.99
AD-106	LC	72	M	18	0.9	4.3	103	18.7	5.2	1.83
AD-138	LC	77	F	4	0.7	4	88	8.6	5.5	5.69
AD-148	LC	88	F	21	1.1	3.6	84	9.3	7.4	7.86
AD-151	LC	73	F	11	0.4	4	97	9.1	5.1	1.92
SL-15	CH	66	F	56	0.7	4	96	17.7	71.9	4.37
SL-46	LC	65	F	22	0.7	3.7	72	8.2	19.5	3.721
SL-121	CH	81	M	17	0.9	3.8	92	13.5	4.7	NT
SL-137	CH	72	F	44	0.8	3.6	94	15	13.5	1.23
SL-151	LC	46	M	54	1.3	2.8	58	6.8	55.2	5.16
SL-158	LC	78	F	38	0.8	3.8	66	9.2	22.5	6.67
SL-167	CH	79	M	21	0.5	3.2	81	24.6	2.6	2.11
SL-248	CH	71	M	18	1.3	4.1	93	10.5	5.4	0.91
SL-252	LC	82	F	26	1.3	3.4	80	3.8	9.4	9.36

SR-6	LC	69	M	17	0.6	3.7	40	8.8	9.1	8.33
SR-14	LC	72	M	41	0.7	3.6	75	18.8	NT	NT
SR-23	CH	74	M	12	0.7	4.3	80	19.5	3.4	0.84
SR-80	LC	76	F	21	1.7	3.8	84	14.8	7.9	NT
SR-103	CH	85	M	7	1.4	3.9	80	18.2	5.7	NT
SR-158	LC	74	M	40	1	3.9	91	11.6	NT	NT

Abbreviation, HCC; onset data of HCC after DAA treatment, SVR; achieve date of SVR 12.

Table S2. Result of each individual HCC prediction.

A. Result of HCC recurrence prediction in individual liver cirrhosis case

	4 miRNAs			3 miRNAs			2 miRNAs		
**Case**	**value**	**Prediction**	**FALSE**	**value**	**Prediction**	**FALSE**	**value**	**Prediction**	**FALSE**
AD-10	-1.94	N		-2.11	N		0.01	T	*
AD-44	-7.93	N		-7.09	N		-2.20	N	
AD-45	-3.77	N		-4.27	N		-1.07	N	
AD-53	-4.19	N		-4.10	N		-0.83	N	
AD-92	4.20	T	*	4.23	T	*	2.17	T	*
AD-109	-6.16	N		-6.24	N		-1.87	N	
AD-117	-2.98	N		-3.72	N		-1.41	N	
AD-136	-1.00	N		-0.30	N		0.35	T	*
SL-2	-1.00	N		-1.71	N		-0.55	N	
SL-6	-0.63	N		-0.44	N		-0.38	N	
SL-20	-4.09	N		-4.00	N		-0.70	N	
SL-40	-3.99	N		-5.42	N		-1.83	N	
SL-87	-1.10	N		-1.00	N		-1.00	N	
SL-90	-20.40	N		-37.13	N		-8.21	N	
SL-92	-2.17	N		-1.56	N		-0.04	N	
SL-117	-7.55	N		-9.74	N		-3.18	N	
SL-132	-4.74	N		-5.95	N		-2.52	N	
SL-205	-3.39	N		-4.12	N		-1.05	N	
SL-224	-10.60	N		-12.33	N		-3.19	N	
SL-228	-7.72	N		-8.84	N		-2.15	N	
SL-257	-3.31	N		-3.55	N		-1.55	N	
SL-263	-16.55	N		-19.62	N		-3.70	N	
SL-272	-2.77	N		-4.35	N		-1.37	N	
SR-53	-0.86	N		-0.18	N		0.98	T	*
SR-168	-2.83	N		-3.27	N		-1.01	N	
AD-22	1.93	T		1.78	T		1.16	T	
AD-24	3.93	T		3.65	T		2.01	T	
AD-47	1.03	T		1.98	T		-0.18	N	*
AD-75	7.29	T		7.29	T		2.10	T	
AD-106	1.00	T		0.32	T		0.99	T	
AD-138	1.46	T		2.94	T		0.07	T	
AD-148	4.21	T		4.19	T		1.97	T	
AD-151	0.21	T		1.00	T		0.47	T	
SL-46	4.55	T		4.52	T		2.56	T	
SL-151	3.48	T		2.74	T		1.07	T	
SL-158	1.00	T		0.99	T		0.93	T	
SL-252	5.79	T		7.51	T		1.37	T	
SR-6	0.99	T		2.11	T		-1.35	N	*
SR-14	1.02	T		1.00	T		-0.34	N	*
SR-80	3.32	T		3.18	T		1.57	T	
SR-158	2.74	T		3.41	T		1.52	T	

B. Result of HCC recurrence prediction in individual case

	4 miRNAs			3 miRNAs			2 miRNAs		
**case**	**value**	**prediction**	**FALSE**	**value**	**prediction**	**FALSE**	**value**	**prediction**	**FALSE**
AD-10	-0.26	N		-0.08	N		0.27	T	*
AD-40	-1.81	N		-1.78	N		-1.67	N	
AD-44	-3.04	N		-1.82	N		-1.38	N	
AD-45	-2.23	N		-2.20	N		-1.90	N	
AD-49	-2.43	N		-1.62	N		-1.20	N	
AD-50	-1.94	N		-1.28	N		-0.88	N	

AD-53	-1.51	N		-0.83	N		-0.62	N	
AD-67	-2.07	N		-2.21	N		-1.63	N	
AD-92	0.17	T	*	-0.78	T		-0.93	T	
AD-109	-1.35	N		-0.45	N		-0.11	N	
AD-111	-1.07	N		-0.61	N		-0.38	N	
AD-117	-0.60	N		-0.04	N		-0.20	N	
AD-120	-0.99	N		-0.48	N		-0.26	N	
AD-136	-0.94	N		-0.53	N		-0.52	N	
AD-150	-0.96	N		-1.35	N		-1.00	N	
SL-2	0.09	T	*	-0.14	T		-0.35	T	
SL-6	-1.28	N		-0.98	N		-1.34	N	
SL-20	0.00	T	*	0.32	T	*	0.45	T	*
SL-40	-1.21	N		-1.05	N		-1.20	N	
SL-52	-1.28	N		-0.03	N		0.14	T	*
SL-82	-1.45	N		-2.79	N		-3.98	N	
SL-87	-3.27	N		-3.70	N		-3.85	N	
SL-90	-1.00	N		-5.71	N		-4.59	N	
SL-92	-2.17	N		-2.16	N		-1.38	N	
SL-101	-1.53	N		-0.09	N		-0.32	N	
SL-106	-0.87	N		-0.75	N		-0.78	N	
SL-117	-1.44	N		-1.26	N		-1.46	N	
SL-132	-2.55	N		-2.51	N		-2.67	N	
SL-152	-1.06	N		-0.68	N		-0.56	N	
SL-161	-0.25	N		-0.54	N		-0.47	N	
SL-198	-1.35	N		-0.56	N		-0.34	N	
SL-205	-0.89	N		-0.93	N		-1.09	N	
SL-224	-1.02	N		-1.00	N		-0.54	N	
SL-228	-1.91	N		-2.21	N		-0.61	N	
SL-234	-0.51	N		-1.36	N		-0.87	N	
SL-257	-1.32	N		-0.34	N		-0.91	N	
SL-263	-2.29	N		-3.02	N		0.53	T	*
SL-272	-0.40	N		-0.43	N		-0.27	N	
SL-294	-4.49	N		-4.69	N		-3.48	N	
SL-301	-1.19	N		-4.91	N		-4.59	N	
SL-323	1.75	T	*	1.77	T	*	1.27	T	*
SR-53	1.61	T	*	1.77	T	*	0.99	T	*
SR-88	1.30	T	*	1.94	T	*	1.77	T	*
SR-168	-3.78	N		-4.91	N		-4.59	N	
AD-22	1.78	T		0.89	T		1.26	T	
AD-24	2.44	T		1.86	T		1.58	T	
AD-47	1.31	T		3.40	T		1.00	T	
AD-54	-3.17	N	*	-3.57	N	*	-2.22	N	*
AD-57	3.84	T		5.56	T		1.37	T	
AD-75	0.98	T		0.99	T		-1.87	N	*
AD-106	0.99	T		0.49	T		0.25	T	
AD-138	2.26	T		3.44	T		3.37	T	
AD-148	2.35	T		1.81	T		1.01	T	
AD-151	-2.15	N	*	-2.79	N	*	-2.31	N	*
SL-15	0.60	T		-0.38	N	*	-0.66	N	*
SL-46	1.14	T		0.27	T		-0.97	N	*
SL-121	0.91	T		1.21	T		1.05	T	
SL-137	0.37	T		0.42	T		0.57	T	
SL-151	1.02	T		0.29	T		0.53	T	
SL-158	-0.40	N	*	-0.75	N	*	-0.74	N	*
SL-167	1.90	T		0.98	T		0.58	T	
SL-248	2.03	T		1.48	T		1.32	T	

SL-252	1.46	T		1.65	T		1.43	T	
SR-6	0.44	T		1.00	T		0.87	T	
SR-14	1.88	T		2.57	T		1.54	T	
SR-23	0.62	T		0.62	T		0.57	T	
SR-80	1.16	T		0.52	T		0.24	T	
SR-103	2.89	T		2.94	T		1.19	T	
SR-158	1.24	T		0.91	T		0.98	T	

C. Result of HCC occurrence prediction in individual case

	**4 miRNAs**			**3 miRNAs**			2 miRNAs		
**case**	**value**	**prediction**	**FALSE**	**value**	**Prediction**	**FALSE**	**value**	**prediction**	**FALSE**
AD-2	-1.27	N		-1.13	N		-1.10	N	
AD-3	-1.51	N		-1.00	N		-1.01	N	
AD-5	-1.47	N		-1.04	N		-1.11	N	
AD-6	-1.08	N		-1.05	N		-1.00	N	
AD-11	-1.41	N		-1.29	N		-1.04	N	
AD-12	0.02	T	*	-0.41	N		-0.11	N	
AD-27	-1.00	N		-1.20	N		-1.04	N	
AD-33	-2.20	N		-1.00	N		-0.89	N	
AD-36	-1.32	N		-0.99	N		-1.07	N	
AD-38	-1.00	N		-1.00	N		-0.99	N	
AD-39	-1.12	N		-0.98	N		-1.08	N	
AD-51	-1.23	N		-0.99	N		-1.12	N	
AD-52	-1.14	N		-1.19	N		-1.04	N	
AD-56	-0.83	N		-0.99	N		-1.05	N	
AD-58	-1.30	N		-1.14	N		-1.08	N	
AD-70	0-.69	N		-0.79	N		-0.81	N	
AD-82	-0.73	N		-1.16	N		-0.93	N	
AD-84	-1.01	N		-1.00	N		-1.01	N	
AD-94	-1.00	N		-1.08	N		-1.01	N	
AD-96	-1.00	N		-1.00	N		-0.94	N	
AD-97	-1.00	N		-0.99	N		-0.93	N	
AD-104	-1.00	N		-1.00	N		-1.03	N	
AD-105	-0.90	N		-1.00	N		-1.05	N	
AD-113	-1.33	N		-1.02	N		-1.12	N	
AD-122	-1.00	N		-1.17	N		-1.12	N	
AD-123	-1.03	N		-1.04	N		-1.06	N	
AD-129	-2.40	N		-1.01	N		-1.03	N	
AD-131	-1.29	N		-1.05	N		-1.06	N	
AD-132	-1.36	N		-1.28	N		-1.08	N	
AD-137	-1.59	N		-0.99	N		-1.09	N	
AD-142	-2.66	N		-1.00	N		-0.99	N	
AD-154	-1.42	N		-0.96	N		-1.00	N	
SL-27	-1.00	N		-1.10	N		-1.04	N	
SL-49	-1.41	N		-1.00	N		-1.08	N	
SL-65	-1.32	N		-0.74	N		-0.04	N	
SL-108	-0.71	N		0.11	T	*	0.03	T	*
SL-162	-0.59	N		0.14	T	*	0.07	T	*
SL-208	-0.24	N		-2.71	N		-0.60	N	
SL-99	-1.00	N		-0.57	N		-0.22	N	
SL-220	-0.47	N		-0.44	N		-0.60	N	
SL-226	-2.96	N		-0.27	N		-0.25	N	
SL-268	-1.84	N		-0.68	N		-0.24	N	
SL-284	-1.22	N		-0.91	N		-0.33	N	
SR-8	-1.73	N		-3.02	N		-0.77	N	
SR-59	0.29	T	*	-0.01	N		0.19	T	*

SR-81	-2.50	N		-1.00	N		-0.06	N	
SL-154	-1.39	N		-1.30	N		-0.96	N	
SL-155	-0.85	N		-0.20	N		0.82	T	*
SL-157	-1.00	N		-1.03	N		-1.00	N	
SL-159	0.75	T	*	-0.96	N		0.46	T	*
SL-160	0.15	T	*	-1.06	N		-1.00	N	
SL-163	-2.63	N		-0.27	N		-0.13	N	
SL-165	-3.80	N		-0.89	N		-1.00	N	
SL-166	-1.02	N		-2.09	N		1.73	T	*
SL-168	-1.67	N		-0.91	N		-0.26	N	
AD-1	1.00	T		1.01	T		1.00	T	
AD-7	-1.09	N	*	-1.05	N	*	-1.12	N	*
AD-26	8.02	T		1.17	T		1.00	T	
AD-59	1.45	T		0.20	T		0.23	T	
AD-116	-1.06	N	*	-0.99	N	*	-1.07	N	*
SL-35	1.55	T		1.00	T		1.14	T	
SL-44	3.67	T		1.04	T		1.13	T	
SL-48	1.00	T		1.28	T		1.24	T	
SL-153	1.00	T		-0.95	N	*	-1.07	N	*
SL-173	1.00	T		0.21	T		0.20	T	
SL-192	1.00	T		1.05	T		1.54	T	
SL-293	1.00	T		0.87	T		1.00	T	
SL-341	7.02	T		-1.16	N	*	-1.09	N	*
SR-3	4.22	T		2.02	T		2.42	T	
SR-16	1.44	T		1.12	T		1.78	T	

Abbreviations. value; values by each calculation formula, prediction T; HCC development, N; HCC non-development, FALSE *; prediction did not match result.

Table S3. List of exosomal miRNAs in liver cirrhosis that were significantly differentially expressed between recurring-HCC and non-recurring HCC groups. ($p < 0.05$).

miRNA	ratio	*p*-value	miRNA	ratio	*p*-value
hsa-miR-3973	0.43	1.06E-03	hsa-miR-6881-3p	1.50	3.60E-02
hsa-miR-4802-5p	0.51	9.42E-03	hsa-miR-4639-3p	1.52	3.87E-02
hsa-miR-4802-3p	0.54	1.43E-02	hsa-miR-4441	1.53	3.18E-02
hsa-miR-3936	0.54	9.95E-03	hsa-miR-181b-5p	1.54	2.66E-02
hsa-miR-4704-5p	0.55	3.88E-02	hsa-miR-3074-5p	1.54	4.12E-02
hsa-miR-1178-3p	0.58	3.07E-02	hsa-miR-6740-5p	1.55	2.98E-02
hsa-miR-1270	0.58	3.27E-02	hsa-miR-24-2-5p	1.56	4.10E-02
hsa-miR-199a-3p, hsa-miR-199b-3p	0.60	4.49E-02	hsa-miR-1287-5p	1.56	4.45E-02
hsa-miR-3186-3p	0.62	4.85E-02	hsa-miR-6728-5p	1.56	2.63E-02
hsa-miR-6758-3p	0.63	3.30E-02	hsa-miR-4420	1.57	3.70E-02
hsa-miR-5589-3p	0.63	2.63E-02	hsa-miR-3155b	1.58	1.95E-02
hsa-miR-6807-3p	0.70	2.39E-02	hsa-miR-29c-5p	1.59	2.25E-02
hsa-miR-3688-5p	0.76	3.71E-02	hsa-miR-501-5p	1.60	2.07E-02
hsa-miR-4659b-3p	0.79	3.27E-02	hsa-miR-4329	1.61	3.31E-02
hsa-miR-4515	1.18	2.61E-02	hsa-miR-921	1.63	3.59E-02
hsa-miR-4718	1.20	6.49E-04	hsa-miR-7854-3p	1.63	4.11E-02
hsa-miR-4755-3p	1.27	2.20E-02	hsa-miR-4711-3p	1.64	3.79E-02
hsa-miR-4308	1.34	4.86E-02	hsa-miR-3591-3p	1.66	3.68E-02
hsa-miR-7-2-3p	1.36	3.06E-02	hsa-miR-744-3p	1.66	1.00E-02
hsa-miR-6761-3p	1.38	3.71E-02	hsa-miR-6874-5p	1.67	2.88E-02
hsa-miR-4252	1.38	4.55E-02	hsa-miR-1306-3p	1.71	1.23E-02
hsa-miR-6809-3p	1.39	2.56E-02	hsa-miR-378i	1.72	7.19E-03
hsa-miR-4633-3p	1.41	3.86E-02	hsa-miR-4451	1.77	1.52E-02
hsa-miR-519d-3p	1.46	4.64E-02	hsa-miR-222-5p	1.77	2.40E-02
hsa-miR-34a-5p	1.47	4.99E-02	hsa-miR-4474-3p	1.84	3.78E-02
hsa-miR-595	1.49	3.99E-02	hsa-miR-5093	1.85	2.32E-02
hsa-miR-5188	1.49	1.41E-02	hsa-miR-4686	1.97	1.94E-02
hsa-miR-5188	1.49	1.41E-02	hsa-miR-656-5p	2.03	1.70E-02

Abbreviation: ratio; expression ratio of miRNA in HCC no recurrence/recurrence.

Table S4. List of exosomal miRNAs that were expressed significantly different between recurring HCC and non-recurring HCC in non-cirrhosis and liver cirrhosis (p <0.05).

miRNA	ratio	*p*-value	miRNA	ratio	*p*-value
hsa-miR-3973	0.54	1.04E-03	hsa-miR-3150b-5p	0.83	3.70E-02
hsa-miR-6758-3p	0.57	1.29E-03	hsa-miR-4299	0.84	4.55E-02
hsa-miR-3936	0.58	3.74E-03	hsa-miR-4659b-3p	0.85	4.49E-02
hsa-miR-1178-3p	0.59	6.29E-03	hsa-miR-548w	0.85	3.30E-02
hsa-miR-4802-5p	0.63	1.90E-02	hsa-miR-548h-5p	0.86	2.89E-02
hsa-miR-5584-3p	0.63	2.47E-02	hsa-miR-548aw	0.88	4.73E-02
hsa-miR-4501	0.63	2.71E-02	hsa-miR-204-3p	1.11	3.39E-02
hsa-miR-199a-3p, hsa-miR-199b-3p	0.65	2.20E-02	hsa-miR-4448	1.12	2.94E-02
hsa-miR-3186-3p	0.65	1.65E-02	hsa-miR-4515	1.16	2.32E-02
hsa-miR-4802-3p	0.65	2.51E-02	hsa-miR-4668-3p	1.18	4.91E-02
hsa-miR-4801	0.65	3.39E-02	hsa-miR-3194-3p	1.18	3.97E-03
hsa-miR-1208	0.65	4.16E-02	hsa-miR-4718	1.20	2.53E-05
hsa-miR-5004-3p	0.66	1.86E-02	hsa-miR-3160-5p	1.20	1.09E-02
hsa-miR-1255b-2-3p	0.66	3.99E-02	hsa-miR-379-3p	1.21	4.93E-02
hsa-miR-1298-5p	0.66	4.85E-02	hsa-miR-4755-3p	1.22	1.68E-02
hsa-miR-203a-5p	0.67	3.19E-02	hsa-miR-3689d	1.31	1.65E-02
hsa-miR-3681-3p	0.67	3.51E-02	hsa-miR-26a-1-3p	1.32	3.46E-02
hsa-miR-135b-5p	0.67	4.65E-02	hsa-miR-5699-3p	1.32	4.04E-02
hsa-miR-1269a	0.68	4.66E-02	hsa-miR-381-3p	1.33	1.43E-02
hsa-miR-1270	0.68	4.17E-02	hsa-miR-181b-5p	1.34	4.83E-02
hsa-miR-3713	0.68	3.13E-02	hsa-miR-29c-5p	1.35	4.65E-02
hsa-miR-3130-3p	0.69	4.41E-02	hsa-miR-4727-3p	1.36	1.58E-02
hsa-miR-3651	0.72	4.15E-02	hsa-miR-519b-3p	1.36	2.69E-02
hsa-miR-6821-3p	0.73	3.45E-02	hsa-miR-579-3p	1.37	1.35E-02
hsa-miR-6070	0.74	7.85E-03	hsa-miR-22-5p	1.38	1.88E-02
hsa-miR-6864-3p	0.75	1.93E-02	hsa-miR-1287-5p	1.38	4.58E-02
hsa-miR-98-3p	0.76	3.25E-02	hsa-miR-595	1.38	3.15E-02
hsa-miR-32-3p	0.77	1.28E-02	hsa-miR-769-3p	1.40	3.79E-02
hsa-miR-5197-5p	0.77	4.49E-02	hsa-miR-3123	1.41	2.13E-02
hsa-miR-6807-3p	0.79	2.45E-02	hsa-miR-3074-5p	1.41	2.33E-02
hsa-miR-4330	0.80	4.83E-02	hsa-miR-3591-3p	1.44	3.41E-02
hsa-miR-623	0.81	7.00E-03	hsa-miR-4633-3p	1.45	3.68E-03
hsa-miR-99b-5p	0.81	4.98E-02	hsa-miR-7157-5p	1.46	3.29E-02
hsa-miR-3688-5p	0.83	2.78E-02	hsa-miR-5188	1.58	2.11E-03
hsa-miR-3688-5p	0.83	2.78E-02	hsa-miR-656-5p	1.64	2.76E-02

Abbreviation: ratio; expression ratio of miRNA in HCC no recurrence/recurrence.

Table S5. The accuracy of predicting recurring HCC using single exosomal miRNA expression.

liver cirrhosis		non-cirrhosis and liver cirrhosis	
**miRNA**	**prediction rate**	**miRNA**	**prediction rate**
hsa-miR-4268	0.60	hsa-miR-550a-5p	0.60
hsa-miR-2467-3p	0.62	hsa-miR-2467-3p	0.61
hsa-miR-4708-3p	0.63	hsa-miR-204-3p	0.62
hsa-miR-204-3p	0.63	hsa-miR-6826-3p	0.63
hsa-miR-4755-3p	0.65	hsa-miR-4755-3p	0.63
hsa-miR-4515	0.65	hsa-miR-4740-5p	0.67
hsa-miR-3160-5p	0.67	hsa-miR-3194-3p	0.69
hsa-miR-3194-3p	0.69	hsa-miR-4515	0.69
hsa-miR-4448	0.74	hsa-miR-3160-5p	0.72
hsa-miR-4718	0.76	hsa-miR-4448	0.73
		hsa-miR-4718	0.73

Table S6. List of exosomal miRNAs with expression that were significantly different between non-recurring HCC and first time occurring HCC groups ($p < 0.05$).

mRNA	ratio	*p*-value	miRNA	ratio	*p*-value
hsa-miR-4663	0.56	6.75E-05	hsa-miR-6880-3p	1.19	4.22E-02
hsa-miR-17-5p	0.59	1.10E-03	hsa-miR-4530	1.20	8.08E-03
hsa-miR-106a-5p	0.61	5.32E-03	hsa-miR-150-3p	1.20	3.40E-02
hsa-miR-4528	0.61	5.57E-03	hsa-miR-3679-5p	1.20	1.58E-02
hsa-miR-29b-3p	0.62	1.09E-03	hsa-miR-4294	1.20	1.36E-02
hsa-miR-21-5p	0.63	2.09E-03	hsa-miR-744-5p	1.20	3.39E-02
hsa-miR-3194-3p	0.63	1.28E-03	hsa-miR-7111-5p	1.20	4.01E-02
hsa-miR-126-3p	0.63	4.25E-03	hsa-miR-6819-5p	1.20	1.98E-02
hsa-miR-20a-5p	0.63	1.02E-02	hsa-miR-6769a-5p	1.20	4.01E-02
hsa-miR-6073	0.63	2.20E-03	hsa-miR-1225-5p	1.20	3.71E-02
hsa-let-7a-5p	0.64	3.26E-03	hsa-miR-4459	1.21	1.51E-02
hsa-miR-130a-3p	0.65	1.72E-02	hsa-miR-6795-3p	1.21	4.79E-02
hsa-miR-4317	0.65	1.22E-02	hsa-miR-6799-5p	1.21	1.45E-02
hsa-let-7f-5p	0.66	2.34E-02	hsa-miR-6802-5p	1.21	1.51E-02
hsa-miR-26a-5p	0.66	6.73E-03	hsa-miR-373-5p	1.21	3.77E-02
hsa-miR-4755-3p	0.66	1.92E-02	hsa-miR-6800-3p	1.22	4.08E-02
hsa-miR-20b-5p	0.67	1.84E-02	hsa-miR-4486	1.22	1.73E-02
hsa-miR-107	0.67	2.79E-03	hsa-miR-6768-5p	1.22	2.82E-03
hsa-let-7d-5p	0.67	7.06E-03	hsa-miR-5008-5p	1.22	4.48E-02
hsa-miR-4423-5p	0.68	4.06E-02	hsa-miR-937-5p	1.23	1.21E-02
hsa-miR-28-5p	0.69	1.35E-02	hsa-miR-4298	1.23	4.95E-02
hsa-miR-100-5p	0.69	2.92E-02	hsa-miR-6824-5p	1.23	4.77E-02
hsa-miR-17-3p	0.69	1.37E-02	hsa-miR-6732-3p	1.23	4.89E-02
hsa-miR-16-5p	0.69	1.35E-02	hsa-miR-6756-5p	1.23	8.48E-03
hsa-miR-4694-5p	0.69	4.86E-03	hsa-miR-6826-5p	1.23	4.97E-02
hsa-miR-10b-5p	0.69	2.43E-02	hsa-miR-3679-3p	1.24	1.90E-02
hsa-miR-15a-5p	0.69	1.46E-02	hsa-miR-6798-3p	1.24	2.36E-02
hsa-miR-30c-5p	0.69	1.78E-02	hsa-miR-197-5p	1.24	1.03E-02
hsa-miR-524-5p	0.69	1.70E-03	hsa-miR-6746-5p	1.24	4.64E-02
hsa-miR-4325	0.69	3.04E-02	hsa-miR-6132	1.24	1.96E-02
hsa-miR-5009-5p	0.70	4.30E-02	hsa-miR-4447	1.24	3.81E-02
hsa-miR-24-1-5p	0.70	1.97E-03	hsa-miR-625-3p	1.24	1.04E-02
hsa-let-7g-5p	0.70	3.44E-02	hsa-miR-1238-3p	1.25	3.39E-02
hsa-miR-153-5p	0.70	1.16E-02	hsa-miR-6880-5p	1.25	6.72E-03
hsa-miR-4536-3p	0.70	1.26E-02	hsa-miR-6845-3p	1.25	4.29E-02
hsa-miR-4515	0.70	1.67E-02	hsa-miR-6891-3p	1.25	3.40E-02
hsa-miR-363-3p	0.70	3.90E-03	hsa-miR-4443	1.25	4.59E-02
hsa-miR-342-5p	0.71	1.80E-02	hsa-miR-5195-3p	1.26	1.36E-02
hsa-miR-525-3p	0.71	3.12E-03	hsa-miR-4731-3p	1.26	1.46E-02
hsa-miR-4460	0.71	1.47E-02	hsa-miR-6797-5p	1.26	4.70E-02
hsa-let-7c-5p	0.71	3.13E-02	hsa-miR-4513	1.26	4.83E-02
hsa-miR-4771	0.71	4.99E-02	hsa-miR-4783-3p	1.27	3.77E-02
hsa-miR-4480	0.71	4.40E-02	hsa-miR-6825-5p	1.27	4.75E-02
hsa-miR-25-3p	0.72	3.43E-02	hsa-miR-6889-5p	1.27	4.02E-02
hsa-miR-18a-5p	0.72	5.48E-03	hsa-miR-6076	1.27	4.56E-02
hsa-miR-27a-3p	0.72	3.61E-02	hsa-miR-6779-5p	1.27	2.51E-02
hsa-miR-520d-5p	0.72	1.67E-02	hsa-miR-557	1.28	6.40E-03
hsa-miR-103a-3p	0.72	1.33E-02	hsa-miR-6813-5p	1.28	4.36E-02
hsa-miR-518e-3p	0.72	2.91E-02	hsa-miR-6865-3p	1.28	3.76E-02
hsa-miR-652-3p	0.72	3.63E-02	hsa-miR-8089	1.28	3.15E-02
hsa-miR-492	0.73	3.56E-03	hsa-miR-6826-3p	1.28	4.99E-02
hsa-miR-640	0.73	1.07E-02	hsa-miR-4750-5p	1.29	3.49E-02
hsa-miR-101-3p	0.73	3.19E-03	hsa-miR-652-5p	1.29	2.83E-02

hsa-miR-143-3p	0.73	2.26E-02	hsa-miR-7109-5p	1.29	1.03E-02
hsa-miR-99a-5p	0.73	4.81E-02	hsa-miR-6757-5p	1.29	1.02E-02
hsa-miR-1911-5p	0.74	4.12E-03	hsa-miR-7155-5p	1.29	4.75E-02
hsa-miR-221-3p	0.74	2.61E-02	hsa-miR-6803-3p	1.30	4.38E-02
hsa-miR-563	0.75	1.51E-03	hsa-miR-4483	1.30	3.44E-02
hsa-miR-576-5p	0.75	2.42E-02	hsa-miR-4419b	1.30	4.54E-02
hsa-miR-519d-5p	0.75	2.12E-02	hsa-miR-6769b-5p	1.30	3.34E-02
hsa-miR-520f-5p	0.75	1.18E-02	hsa-miR-642a-3p	1.30	1.07E-02
hsa-miR-132-3p	0.76	4.27E-02	hsa-miR-6782-5p	1.30	4.74E-02
hsa-miR-490-5p	0.76	2.05E-02	hsa-miR-6735-5p	1.31	4.44E-02
hsa-miR-651-5p	0.76	4.22E-03	hsa-miR-8485	1.31	4.14E-02
hsa-miR-29b-1-5p	0.76	4.51E-02	hsa-miR-6784-3p	1.31	3.33E-02
hsa-miR-4650-3p	0.77	2.56E-03	hsa-miR-6716-5p	1.31	2.22E-02
hsa-miR-4666a-3p	0.77	5.41E-04	hsa-miR-6859-3p	1.31	2.27E-02
hsa-miR-382-3p	0.78	1.03E-02	hsa-miR-3188	1.32	2.13E-02
hsa-miR-223-3p	0.78	1.21E-02	hsa-miR-7847-3p	1.33	1.27E-02
hsa-miR-629-5p	0.78	2.02E-02	hsa-miR-4476	1.34	6.41E-03
hsa-miR-124-3p	0.78	4.12E-02	hsa-miR-4685-5p	1.34	2.09E-02
hsa-miR-1256	0.79	3.59E-02	hsa-miR-3154	1.34	2.19E-02
hsa-miR-548e-3p	0.79	3.76E-02	hsa-miR-6848-3p	1.35	3.08E-02
hsa-miR-451a	0.79	1.73E-02	hsa-miR-6790-3p	1.35	1.45E-02
hsa-miR-548aq-5p	0.80	1.54E-02	hsa-miR-6802-3p	1.35	4.09E-02
hsa-miR-548ar-5p	0.80	1.13E-02	hsa-miR-1185-1-3p	1.35	4.65E-02
hsa-miR-496	0.80	4.48E-02	hsa-miR-8071	1.35	1.66E-02
hsa-miR-1279	0.80	1.76E-02	hsa-miR-7846-3p	1.36	1.04E-02
hsa-miR-586	0.80	2.55E-02	hsa-miR-6740-3p	1.36	4.81E-02
hsa-miR-218-2-3p	0.80	1.74E-02	hsa-miR-575	1.36	3.16E-02
hsa-miR-299-3p	0.81	1.48E-02	hsa-miR-671-5p	1.36	7.41E-03
hsa-miR-191-5p	0.81	4.34E-02	hsa-miR-6511a-3p	1.36	3.76E-02
hsa-miR-1290	0.81	1.28E-02	hsa-miR-4526	1.36	1.73E-02
hsa-miR-4522	0.81	4.49E-02	hsa-miR-6895-5p	1.36	4.94E-02
hsa-miR-9-3p	0.81	3.96E-02	hsa-miR-564	1.36	1.69E-02
hsa-miR-571	0.81	5.10E-03	hsa-miR-6846-3p	1.37	4.69E-02
hsa-miR-4491	0.81	4.49E-02	hsa-miR-4747-5p	1.37	4.65E-02
hsa-miR-6131	0.82	2.39E-03	hsa-miR-6829-3p	1.37	3.59E-02
hsa-miR-4520-2-3p	0.83	3.60E-02	hsa-miR-6842-5p	1.38	1.54E-02
hsa-miR-30e-3p	0.83	1.46E-02	hsa-miR-1296-3p	1.38	3.68E-02
hsa-miR-3121-5p	0.84	1.79E-02	hsa-miR-6790-5p	1.38	2.95E-02
hsa-miR-4309	0.84	2.85E-02	hsa-miR-3175	1.39	2.32E-02
hsa-miR-23b-3p	0.84	2.38E-02	hsa-miR-6857-3p	1.41	3.90E-02
hsa-miR-92b-3p	0.85	2.10E-02	hsa-miR-1185-2-3p	1.41	3.83E-02
hsa-miR-5092	0.85	2.07E-02	hsa-miR-6807-5p	1.42	1.01E-02
hsa-miR-4469	0.85	3.35E-02	hsa-miR-6832-3p	1.42	3.39E-02
hsa-miR-153-3p	0.85	4.49E-02	hsa-miR-6871-5p	1.42	4.91E-02
hsa-miR-3139	0.85	2.66E-02	hsa-miR-4419a	1.43	1.79E-02
hsa-miR-23a-3p	0.85	3.47E-02	hsa-miR-6866-3p	1.43	4.14E-02
hsa-miR-1246	0.86	1.36E-02	hsa-miR-489-5p	1.43	4.99E-02
hsa-miR-802	0.87	5.52E-03	hsa-miR-6778-3p	1.44	3.16E-02
hsa-miR-3688-5p	0.88	7.23E-03	hsa-miR-6830-5p	1.44	4.41E-02
hsa-miR-577	0.88	7.17E-03	hsa-miR-885-3p	1.44	4.59E-02
hsa-miR-1178-5p	0.88	3.26E-02	hsa-miR-664b-3p	1.44	3.73E-02
hsa-miR-4729	0.88	2.97E-02	hsa-miR-6746-3p	1.44	3.52E-02
hsa-miR-374b-5p	0.88	1.38E-02	hsa-miR-4329	1.45	4.40E-02
hsa-miR-3200-5p	0.89	1.96E-02	hsa-miR-4740-3p	1.46	2.03E-02
hsa-miR-3664-5p	0.89	8.70E-03	hsa-miR-6747-5p	1.47	4.54E-02
hsa-miR-548f-5p	0.90	5.61E-03	hsa-miR-1226-3p	1.47	3.08E-02

hsa-miR-548c-3p	0.90	4.06E-02	hsa-miR-7113-5p	1.47	4.38E-02
hsa-miR-613	0.90	9.75E-03	hsa-miR-5685	1.48	4.40E-02
hsa-miR-614	0.91	3.98E-03	hsa-miR-3911	1.48	2.85E-02
hsa-miR-4426	0.92	4.98E-02	hsa-miR-519d-3p	1.48	4.41E-02
hsa-miR-302c-3p	0.93	3.19E-02	hsa-miR-6835-5p	1.49	1.65E-02
hsa-miR-4799-3p	0.93	4.88E-02	hsa-miR-4481	1.49	1.57E-02
hsa-miR-4746-5p	0.94	4.81E-02	hsa-miR-6841-3p	1.49	4.40E-02
hsa-miR-4258	0.94	4.74E-02	hsa-miR-3610	1.49	3.54E-02
hsa-miR-4677-3p	0.98	5.85E-03	hsa-miR-6874-5p	1.50	4.46E-02
hsa-miR-664a-5p	1.03	4.74E-02	hsa-miR-6831-5p	1.50	7.95E-03
hsa-miR-1237-5p	1.07	4.52E-02	hsa-miR-6878-3p	1.50	4.45E-02
hsa-miR-6869-5p	1.07	4.73E-02	hsa-miR-6828-5p	1.50	4.59E-02
hsa-miR-3940-5p	1.07	4.24E-02	hsa-miR-4717-3p	1.50	4.27E-02
hsa-miR-6087	1.08	4.98E-02	hsa-miR-6836-5p	1.51	1.11E-02
hsa-miR-6126	1.08	2.97E-02	hsa-miR-6833-5p	1.51	4.10E-02
hsa-miR-1228-5p	1.09	4.29E-02	hsa-miR-6873-5p	1.52	4.56E-02
hsa-miR-6752-5p	1.09	3.63E-02	hsa-miR-7702	1.52	4.13E-02
hsa-miR-6816-5p	1.09	3.77E-02	hsa-miR-6830-3p	1.52	2.04E-02
hsa-miR-3621	1.10	2.03E-02	hsa-miR-1236-5p	1.53	2.28E-02
hsa-miR-4467	1.10	4.70E-02	hsa-miR-1303	1.53	3.61E-02
hsa-miR-760	1.10	4.75E-02	hsa-miR-6737-3p	1.53	9.84E-03
hsa-miR-6771-5p	1.10	4.26E-02	hsa-miR-5002-3p	1.54	2.05E-02
hsa-miR-6791-5p	1.11	4.66E-02	hsa-miR-2355-5p	1.54	3.08E-02
hsa-miR-6785-5p	1.11	3.16E-02	hsa-miR-6503-5p	1.54	4.78E-02
hsa-miR-6780b-5p	1.11	3.04E-02	hsa-miR-664a-3p	1.54	4.08E-02
hsa-miR-6088	1.11	3.55E-02	hsa-miR-5587-3p	1.54	3.32E-02
hsa-miR-6798-5p	1.11	3.17E-02	hsa-miR-509-3p	1.54	4.99E-02
hsa-miR-6743-5p	1.11	3.23E-02	hsa-miR-6730-5p	1.55	2.97E-02
hsa-miR-7108-5p	1.12	2.66E-02	hsa-miR-6764-3p	1.55	3.61E-02
hsa-miR-149-3p	1.12	3.31E-02	hsa-miR-4660	1.55	4.23E-02
hsa-miR-1227-5p	1.12	3.64E-02	hsa-miR-1254	1.55	2.36E-02
hsa-miR-328-5p	1.12	2.36E-02	hsa-miR-4686	1.55	4.54E-02
hsa-miR-6085	1.12	2.60E-02	hsa-miR-6854-3p	1.56	4.05E-02
hsa-miR-4505	1.12	4.05E-02	hsa-miR-6742-3p	1.56	1.80E-02
hsa-miR-4463	1.12	2.80E-02	hsa-miR-4506	1.57	2.10E-02
hsa-miR-3180	1.13	4.03E-02	hsa-miR-765	1.57	1.32E-02
hsa-miR-1233-5p	1.13	5.95E-03	hsa-miR-4436a	1.57	3.18E-02
hsa-miR-204-3p	1.13	3.17E-02	hsa-miR-4288	1.57	4.95E-02
hsa-miR-4442	1.13	2.31E-02	hsa-miR-514b-5p	1.58	9.48E-03
hsa-miR-1343-5p	1.13	1.87E-02	hsa-miR-3198	1.58	2.50E-02
hsa-miR-6879-5p	1.14	2.07E-02	hsa-miR-6758-5p	1.58	2.01E-02
hsa-miR-8063	1.14	3.20E-02	hsa-miR-4326	1.58	2.38E-02
hsa-miR-4689	1.14	3.29E-02	hsa-miR-4644	1.58	4.72E-02
hsa-miR-4417	1.14	4.43E-02	hsa-miR-6814-3p	1.59	2.87E-02
hsa-miR-7114-5p	1.14	3.96E-02	hsa-miR-4540	1.59	3.14E-02
hsa-miR-4695-5p	1.15	4.10E-02	hsa-miR-4450	1.60	1.64E-02
hsa-miR-6763-3p	1.15	4.91E-02	hsa-miR-5584-3p	1.60	3.43E-02
hsa-miR-6821-5p	1.15	2.21E-02	hsa-miR-6847-5p	1.60	3.83E-02
hsa-miR-5001-5p	1.15	3.30E-02	hsa-miR-4709-3p	1.61	3.52E-02
hsa-miR-6775-5p	1.15	2.95E-02	hsa-miR-4453	1.61	3.35E-02
hsa-miR-6858-5p	1.15	4.85E-02	hsa-miR-4441	1.63	4.16E-02
hsa-miR-4270	1.15	2.97E-02	hsa-miR-5195-5p	1.64	2.87E-02
hsa-miR-1228-3p	1.15	3.59E-02	hsa-miR-659-3p	1.64	1.08E-02
hsa-miR-296-5p	1.15	3.37E-02	hsa-miR-3678-3p	1.64	2.96E-02
hsa-miR-3195	1.15	2.37E-02	hsa-miR-3713	1.65	4.68E-02
hsa-miR-1238-5p	1.16	1.72E-02	hsa-miR-7848-3p	1.65	1.76E-02

hsa-miR-4632-5p	1.16	2.57E-02	hsa-miR-4451	1.65	3.66E-02
hsa-miR-6800-5p	1.16	2.21E-02	hsa-miR-30b-3p	1.66	2.14E-02
hsa-miR-4687-5p	1.17	3.12E-02	hsa-miR-3667-5p	1.69	4.17E-02
hsa-miR-1249-5p	1.17	4.09E-02	hsa-miR-374c-3p	1.71	2.75E-02
hsa-miR-7150	1.17	4.08E-02	hsa-miR-8055	1.72	1.95E-02
hsa-miR-4792	1.17	3.50E-02	hsa-miR-4474-3p	1.73	1.93E-02
hsa-miR-6796-3p	1.17	4.61E-02	hsa-miR-139-3p	1.75	8.59E-03
hsa-miR-4687-3p	1.17	1.91E-02	hsa-miR-6822-3p	1.76	1.23E-02
hsa-miR-4534	1.17	2.62E-02	hsa-miR-6878-5p	1.78	1.55E-02
hsa-miR-4697-5p	1.17	2.04E-02	hsa-miR-8075	1.78	7.81E-03
hsa-miR-4271	1.17	4.46E-02	hsa-miR-8087	1.78	1.08E-02
hsa-miR-1275	1.18	4.73E-02	hsa-miR-1267	1.80	5.72E-03
hsa-miR-6075	1.18	8.31E-03	hsa-miR-4321	1.80	1.37E-02
hsa-miR-6124	1.18	4.87E-02	hsa-miR-6081	1.80	1.30E-02
hsa-miR-4728-5p	1.18	4.82E-02	hsa-miR-6071	1.81	2.12E-02
hsa-miR-675-5p	1.18	3.81E-02	hsa-miR-3692-5p	1.81	2.20E-02
hsa-miR-6515-3p	1.18	2.29E-02	hsa-miR-873-3p	1.82	1.35E-02
hsa-miR-135a-3p	1.18	4.11E-02	hsa-miR-216b-3p	1.91	7.34E-03
hsa-miR-1229-5p	1.18	2.63E-02	hsa-miR-4682	1.91	3.84E-03
hsa-miR-1202	1.18	4.03E-02	hsa-miR-6757-3p	2.05	3.05E-03
hsa-miR-4433b-5p	1.18	4.16E-02	hsa-miR-4435	2.07	6.91E-03
hsa-miR-4723-5p	1.19	1.38E-02	hsa-miR-6841-5p	2.10	3.78E-03
hsa-miR-6893-5p	1.19	1.62E-02	hsa-miR-433-5p	2.42	1.44E-03
	(1)				

Table S7. List of hepatic miRNAs with expression that were significantly. Different between non-recurring HCC and first time occurring HCC groups ($p < 0.05$).

miRNA	Ratio	p-value	miRNA	Ratio	p-value
hsa-miR-6833-5p	0.52	2.92E-04	hsa-miR-1229-5p	0.85	6.23E-03
hsa-miR-6864-5p	0.52	2.41E-04	hsa-miR-6812-5p	0.85	4.07E-02
hsa-miR-31-3p	0.55	3.59E-03	hsa-miR-4721	0.85	8.81E-03
hsa-miR-1286	0.56	6.26E-04	hsa-miR-5008-5p	0.85	2.63E-03
hsa-miR-659-5p	0.56	2.09E-02	hsa-miR-8085	0.85	1.84E-02
hsa-miR-938	0.57	1.16E-03	hsa-miR-4485-3p	0.85	1.76E-02
hsa-miR-513c-5p	0.57	4.72E-03	hsa-miR-6865-5p	0.85	2.16E-02
hsa-miR-509-3-5p	0.58	4.64E-03	hsa-miR-4696	0.85	7.64E-03
hsa-miR-3667-5p	0.59	3.84E-03	hsa-miR-711	0.85	9.77E-04
hsa-miR-6884-5p	0.59	4.31E-03	hsa-miR-3147	0.85	3.02E-02
hsa-miR-4441	0.60	3.81E-02	hsa-miR-4800-5p	0.85	1.78E-02
hsa-miR-4468	0.60	4.25E-03	hsa-miR-6842-5p	0.86	4.70E-03
hsa-miR-7160-3p	0.60	1.55E-03	hsa-miR-6793-5p	0.86	2.88E-02
hsa-miR-6501-5p	0.61	1.02E-02	hsa-miR-6716-5p	0.86	2.51E-02
hsa-miR-8074	0.61	1.90E-02	hsa-miR-4498	0.86	7.25E-04
hsa-miR-4660	0.62	3.43E-02	hsa-miR-4698	0.86	2.06E-02
hsa-miR-4740-3p	0.62	6.91E-03	hsa-miR-6757-5p	0.86	5.94E-03
hsa-miR-508-5p	0.62	8.58E-03	hsa-miR-5699-5p	0.86	4.41E-03
hsa-miR-4636	0.62	1.03E-03	hsa-miR-1249-5p	0.86	1.61E-03
hsa-miR-767-5p	0.62	1.76E-03	hsa-miR-4999-5p	0.86	3.58E-02
hsa-miR-513b-5p	0.62	3.09E-03	hsa-miR-2392	0.86	4.43E-03
hsa-miR-6758-5p	0.62	2.77E-02	hsa-miR-491-5p	0.86	2.90E-03
hsa-miR-4421	0.63	3.40E-02	hsa-miR-3622a-5p	0.86	1.04E-02
hsa-miR-183-3p	0.63	4.52E-03	hsa-miR-671-5p	0.86	4.36E-03
hsa-miR-3144-5p	0.63	1.14E-03	hsa-miR-365a-5p	0.86	2.20E-02
hsa-miR-1321	0.63	8.69E-03	hsa-miR-4792	0.86	3.99E-03
hsa-miR-7154-3p	0.64	1.26E-03	hsa-miR-642b-3p	0.86	3.80E-03

hsa-miR-4778-5p	0.64	7.39E-04	hsa-miR-8060	0.86	9.42E-03
hsa-miR-6082	0.64	4.24E-02	hsa-miR-4728-5p	0.86	5.83E-03
hsa-miR-6862-5p	0.64	1.06E-02	hsa-miR-3150a-3p	0.86	4.35E-02
hsa-miR-1182	0.64	1.34E-02	hsa-miR-6778-5p	0.86	8.24E-03
hsa-miR-7161-3p	0.64	2.90E-04	hsa-miR-6738-5p	0.86	4.69E-02
hsa-miR-631	0.64	1.99E-05	hsa-miR-6877-5p	0.86	1.84E-03
hsa-miR-3127-5p	0.64	1.55E-02	hsa-miR-3158-5p	0.87	1.65E-02
hsa-miR-3945	0.65	1.01E-02	hsa-miR-3622b-5p	0.87	3.11E-02
hsa-miR-4644	0.65	4.68E-02	hsa-miR-615-3p	0.87	1.70E-02
hsa-miR-526b-3p	0.65	9.70E-03	hsa-miR-6894-5p	0.87	1.04E-03
hsa-miR-873-3p	0.65	2.02E-02	hsa-miR-665	0.87	3.39E-03
hsa-miR-942-3p	0.65	3.98E-03	hsa-miR-3616-3p	0.87	1.28E-03
hsa-miR-4531	0.65	9.77E-04	hsa-miR-483-5p	0.87	4.31E-02
hsa-miR-34c-3p	0.65	1.51E-03	hsa-miR-6806-5p	0.87	3.95E-02
hsa-miR-298	0.65	3.55E-03	hsa-miR-5684	0.87	2.11E-02
hsa-miR-3202	0.66	1.21E-02	hsa-miR-6871-5p	0.88	1.83E-02
hsa-miR-202-3p	0.66	2.00E-02	hsa-miR-1273e	0.88	2.68E-02
hsa-miR-520g-5p	0.66	4.02E-02	hsa-miR-4426	0.88	3.94E-03
hsa-miR-765	0.66	7.94E-04	hsa-miR-1246	0.88	1.40E-02
hsa-miR-1266-3p	0.66	1.51E-02	hsa-miR-1247-3p	0.88	3.08E-03
hsa-miR-3177-5p	0.66	4.64E-03	hsa-miR-6735-5p	0.88	5.00E-03
hsa-miR-3649	0.66	7.03E-03	hsa-miR-933	0.88	2.70E-02
hsa-miR-6874-5p	0.67	1.76E-02	hsa-miR-4430	0.88	8.58E-03
hsa-miR-490-3p	0.67	3.17E-03	hsa-miR-4667-5p	0.88	1.37E-03
hsa-miR-4801	0.67	1.41E-02	hsa-miR-7851-3p	0.88	1.93E-03
hsa-miR-4754	0.67	1.84E-02	hsa-miR-370-3p	0.88	2.84E-03
hsa-miR-4635	0.67	2.76E-03	hsa-miR-1909-5p	0.88	1.09E-02
hsa-miR-4658	0.67	1.37E-02	hsa-miR-6743-5p	0.89	4.44E-03
hsa-miR-4437	0.67	4.78E-02	hsa-miR-3619-3p	0.89	2.69E-02
hsa-miR-4794	0.67	8.54E-03	hsa-miR-7111-5p	0.89	3.82E-02
hsa-miR-219a-2-3p	0.67	1.41E-03	hsa-miR-4299	0.89	3.00E-03
hsa-miR-19a-5p	0.67	4.90E-02	hsa-miR-7107-5p	0.89	1.89E-02
hsa-miR-6770-5p	0.67	1.66E-02	hsa-miR-4485-5p	0.89	3.53E-04
hsa-miR-1236-5p	0.68	2.93E-03	hsa-miR-550a-5p	0.89	3.76E-03
hsa-miR-921	0.68	2.30E-02	hsa-miR-6737-5p	0.89	1.13E-02
hsa-miR-4713-3p	0.68	2.94E-02	hsa-miR-494-3p	0.89	8.75E-03
hsa-miR-6788-5p	0.68	3.28E-02	hsa-miR-6075	0.89	4.82E-03
hsa-miR-1273a	0.69	2.51E-03	hsa-miR-4419a	0.89	2.62E-02
hsa-miR-6835-5p	0.69	3.89E-02	hsa-miR-4673	0.89	3.27E-02
hsa-miR-604	0.69	1.19E-02	hsa-miR-4435	0.89	4.42E-02
hsa-miR-3132	0.69	9.46E-03	hsa-miR-6790-3p	0.90	1.63E-02
hsa-miR-3690	0.69	4.45E-03	hsa-miR-4261	0.90	3.71E-02
hsa-miR-3691-5p	0.70	4.72E-02	hsa-miR-5190	0.90	3.76E-02
hsa-miR-3138	0.70	3.23E-02	hsa-miR-6782-5p	0.90	2.77E-02
hsa-miR-6715b-5p	0.70	4.46E-03	hsa-miR-4459	0.90	1.35E-02
hsa-miR-5581-5p	0.70	6.51E-03	hsa-miR-4449	0.90	4.85E-03
hsa-miR-3161	0.70	7.03E-03	hsa-miR-6722-5p	0.90	8.31E-03
hsa-miR-6833-3p	0.70	2.25E-02	hsa-miR-6132	0.90	3.17E-02
hsa-miR-4470	0.70	4.63E-03	hsa-miR-4726-5p	0.90	2.89E-02
hsa-miR-4300	0.70	4.39E-02	hsa-miR-6750-3p	0.90	4.58E-02
hsa-miR-3153	0.71	3.06E-02	hsa-miR-4656	0.90	7.16E-03
hsa-miR-4436b-3p	0.71	7.10E-03	hsa-miR-4257	0.90	4.07E-02
hsa-miR-4450	0.71	3.04E-03	hsa-miR-7109-5p	0.90	4.92E-03
hsa-miR-3122	0.71	4.27E-03	hsa-miR-3928-3p	0.91	4.06E-02
hsa-miR-548o-3p	0.72	3.54E-02	hsa-miR-4733-3p	0.91	2.33E-02
hsa-miR-4494	0.72	2.86E-02	hsa-miR-1253	0.91	4.71E-02

hsa-miR-4251	0.72	2.79E-02	hsa-miR-6889-5p	0.91	2.48E-02
hsa-miR-6734-3p	0.72	4.42E-02	hsa-miR-4489	0.91	2.15E-02
hsa-miR-6871-3p	0.72	5.15E-03	hsa-miR-3117-3p	0.91	3.32E-02
hsa-miR-1296-3p	0.72	3.01E-03	hsa-miR-1914-3p	0.92	8.78E-03
hsa-miR-299-3p	0.73	1.07E-02	hsa-miR-371a-5p	0.92	2.12E-02
hsa-miR-4785	0.73	3.16E-02	hsa-miR-7641	0.92	4.16E-02
hsa-miR-323a-3p	0.73	6.01E-03	hsa-miR-6780b-5p	0.92	9.51E-03
hsa-miR-1291	0.73	3.31E-02	hsa-miR-8052	0.92	9.64E-03
hsa-miR-6810-5p	0.73	9.85E-03	hsa-miR-7110-5p	0.92	3.42E-02
hsa-miR-4519	0.73	6.82E-03	hsa-miR-4495	0.92	2.43E-02
hsa-miR-6872-5p	0.73	4.89E-02	hsa-miR-3684	0.92	2.14E-02
hsa-miR-7162-3p	0.73	1.29E-02	hsa-miR-1233-5p	0.92	1.73E-03
hsa-miR-363-5p	0.73	5.59E-03	hsa-miR-6769a-5p	0.92	1.45E-02
hsa-miR-3919	0.73	2.47E-02	hsa-miR-6852-5p	0.92	4.42E-02
hsa-miR-5000-3p	0.73	1.34E-02	hsa-miR-4513	0.92	6.43E-03
hsa-miR-6854-5p	0.73	1.28E-02	hsa-miR-4530	0.92	2.73E-02
hsa-miR-3678-3p	0.74	7.08E-03	hsa-miR-1973	0.93	2.37E-03
hsa-miR-4653-3p	0.74	4.31E-03	hsa-miR-1181	0.93	2.71E-02
hsa-miR-3689b-3p, hsa-miR-3689c	0.74	3.34E-02	hsa-miR-6879-5p	0.93	1.81E-02
hsa-miR-4764-5p	0.74	6.86E-03	hsa-miR-4535	0.93	3.86E-02
hsa-miR-6740-5p	0.74	4.89E-02	hsa-miR-6798-5p	0.93	2.11E-02
hsa-miR-2276-3p	0.74	9.06E-03	hsa-miR-642a-3p	0.93	1.95E-02
hsa-miR-6751-3p	0.74	3.05E-02	hsa-miR-3135b	0.93	3.64E-02
hsa-miR-8079	0.74	2.91E-02	hsa-miR-939-5p	0.93	3.28E-02
hsa-miR-30c-1-3p	0.74	1.41E-03	hsa-miR-6763-5p	0.94	2.52E-02
hsa-miR-3976	0.74	2.00E-02	hsa-miR-4749-5p	0.94	1.32E-02
hsa-miR-4303	0.74	1.92E-02	hsa-miR-6808-5p	0.95	4.42E-02
hsa-miR-598-5p	0.74	4.23E-02	hsa-miR-6768-5p	0.95	4.55E-02
hsa-miR-6859-5p	0.74	1.40E-02	hsa-miR-4484	0.95	4.59E-02
hsa-miR-3619-5p	0.74	3.60E-02	hsa-miR-423-5p	0.95	2.85E-02
hsa-miR-6760-5p	0.74	5.33E-03	hsa-miR-6126	0.96	4.50E-02
hsa-miR-650	0.74	4.33E-02	hsa-miR-586	1.02	2.14E-02
hsa-miR-4773	0.74	2.73E-02	hsa-miR-122-5p	1.04	4.75E-02
hsa-miR-5006-5p	0.75	1.20E-03	hsa-miR-4720-3p	1.05	2.14E-02
hsa-miR-4511	0.75	8.06E-03	hsa-miR-548c-3p	1.05	2.33E-02
hsa-miR-6720-3p	0.75	1.88E-02	hsa-miR-5591-5p	1.05	2.14E-02
hsa-miR-1273f	0.75	8.92E-03	hsa-miR-4659a-3p	1.06	2.14E-02
hsa-miR-6510-5p	0.75	4.20E-04	hsa-miR-122-3p	1.06	2.37E-02
hsa-miR-1273c	0.75	4.50E-03	hsa-miR-922	1.07	5.00E-02
hsa-miR-4669	0.75	1.93E-02	hsa-miR-664a-3p	1.08	4.64E-02
hsa-miR-6891-5p	0.75	4.48E-02	hsa-miR-30c-5p	1.08	4.23E-02
hsa-miR-1273h-3p	0.75	2.59E-02	hsa-miR-192-5p	1.09	2.25E-02
hsa-miR-4465	0.76	2.31E-03	hsa-miR-549a	1.09	2.14E-02
hsa-miR-4462	0.76	1.04E-03	hsa-miR-16-5p	1.09	2.42E-02
hsa-miR-550a-3-5p	0.76	2.49E-02	hsa-miR-27b-3p	1.09	4.14E-02
hsa-miR-3944-5p	0.77	1.88E-03	hsa-miR-30b-5p	1.09	2.81E-02
hsa-miR-6769b-5p	0.77	4.05E-04	hsa-miR-100-5p	1.09	4.02E-02
hsa-miR-3682-3p	0.77	1.46E-03	hsa-miR-451a	1.10	2.24E-02
hsa-miR-515-5p	0.77	2.97E-02	hsa-miR-215-5p	1.10	3.05E-02
hsa-miR-7158-5p	0.77	2.47E-02	hsa-let-7g-5p	1.10	3.59E-02
hsa-miR-6830-5p	0.77	1.58E-03	hsa-miR-126-3p	1.10	1.82E-02
hsa-miR-7155-3p	0.77	1.53E-02	hsa-miR-26b-5p	1.11	2.35E-02
hsa-miR-4700-5p	0.78	2.15E-02	hsa-miR-15a-5p	1.11	4.22E-02
hsa-miR-5088-5p	0.78	5.90E-03	hsa-miR-374c-5p	1.12	4.39E-02
hsa-miR-513a-5p	0.78	5.86E-03	hsa-miR-885-5p	1.12	3.32E-02

hsa-miR-4746-5p	0.78	1.67E-02	hsa-miR-6070	1.12	2.00E-02
hsa-miR-3156-5p	0.78	2.40E-02	hsa-miR-3529-3p	1.13	2.70E-02
hsa-miR-4321	0.78	3.66E-03	hsa-miR-223-3p	1.13	3.20E-02
hsa-miR-6837-5p	0.79	1.24E-02	hsa-miR-6807-3p	1.14	1.53E-02
hsa-miR-135a-3p	0.79	2.90E-02	hsa-miR-374a-5p	1.15	4.62E-02
hsa-miR-6807-5p	0.79	1.79E-03	hsa-miR-3167	1.15	2.14E-02
hsa-miR-3714	0.79	2.70E-03	hsa-miR-3913-5p	1.16	2.14E-02
hsa-miR-3934-5p	0.79	8.27E-03	hsa-miR-4744	1.16	4.13E-02
hsa-miR-527, hsa-miR-518a-5p	0.79	1.17E-02	hsa-miR-126-5p	1.16	1.34E-02
hsa-miR-4283	0.79	2.79E-02	hsa-miR-380-5p	1.16	4.90E-02
hsa-miR-4756-5p	0.79	3.42E-03	hsa-miR-335-5p	1.17	2.19E-02
hsa-miR-4732-5p	0.79	1.52E-03	hsa-miR-455-3p	1.17	3.89E-02
hsa-miR-6851-5p	0.79	2.19E-03	hsa-miR-520f-3p	1.18	2.58E-02
hsa-miR-3679-5p	0.79	7.59E-03	hsa-miR-649	1.18	2.10E-02
hsa-miR-6501-3p	0.80	2.62E-03	hsa-miR-4799-3p	1.19	2.14E-02
hsa-miR-4753-5p	0.80	1.51E-03	hsa-miR-7157-3p	1.19	2.00E-02
hsa-miR-4694-3p	0.80	4.18E-02	hsa-miR-4738-5p	1.19	3.27E-02
hsa-miR-4727-3p	0.80	2.19E-02	hsa-miR-587	1.20	3.66E-02
hsa-miR-4757-3p	0.80	4.01E-03	hsa-miR-567	1.20	4.14E-02
hsa-miR-4308	0.80	2.64E-03	hsa-miR-4798-3p	1.20	4.23E-02
hsa-miR-593-5p	0.80	1.21E-02	hsa-miR-548aj-3p	1.20	2.02E-02
hsa-miR-6875-5p	0.80	7.90E-03	hsa-miR-3975	1.21	2.08E-02
hsa-miR-1910-3p	0.80	9.50E-03	hsa-miR-660-5p	1.21	4.87E-02
hsa-miR-9500	0.81	1.06E-03	hsa-miR-6499-3p	1.21	3.35E-02
hsa-miR-6870-5p	0.81	4.97E-03	hsa-miR-6505-3p	1.22	8.69E-03
hsa-miR-6750-5p	0.81	1.46E-03	hsa-miR-548ac	1.22	2.14E-02
hsa-miR-7151-3p	0.81	2.81E-02	hsa-miR-4743-3p	1.22	2.14E-02
hsa-miR-4654	0.81	3.31E-02	hsa-miR-340-5p	1.22	4.88E-02
hsa-miR-4419b	0.81	2.06E-02	hsa-miR-32-5p	1.23	4.07E-02
hsa-miR-6165	0.81	1.85E-03	hsa-miR-4295	1.23	2.09E-02
hsa-miR-4476	0.81	1.10E-03	hsa-miR-4699-3p	1.24	2.30E-02
hsa-miR-5010-5p	0.81	1.59E-02	hsa-miR-609	1.24	4.68E-02
hsa-miR-4428	0.81	1.19E-03	hsa-miR-218-2-3p	1.25	2.14E-02
hsa-miR-4451	0.81	1.46E-02	hsa-miR-1297	1.27	4.93E-02
hsa-miR-3646	0.81	4.49E-02	hsa-miR-2467-5p	1.29	3.33E-02
hsa-miR-1273h-5p	0.81	3.20E-02	hsa-miR-488-3p	1.31	1.82E-03
hsa-miR-6767-5p	0.81	4.14E-02	hsa-miR-517-5p	1.32	1.21E-02
hsa-miR-564	0.81	9.48E-04	hsa-miR-302e	1.32	6.76E-04
hsa-miR-3652	0.81	2.53E-04	hsa-miR-539-5p	1.32	4.36E-02
hsa-miR-3687	0.82	1.04E-02	hsa-miR-708-3p	1.33	4.33E-02
hsa-miR-1254	0.82	1.70E-03	hsa-miR-3142	1.33	1.96E-02
hsa-miR-4294	0.82	1.20E-03	hsa-miR-4708-5p	1.33	2.81E-02
hsa-miR-2116-5p	0.82	3.77E-03	hsa-miR-1247-5p	1.35	4.89E-02
hsa-miR-542-5p	0.82	6.35E-03	hsa-miR-3148	1.35	2.91E-02
hsa-miR-4638-3p	0.82	1.91E-03	hsa-miR-935	1.35	2.69E-02
hsa-miR-652-5p	0.82	2.59E-03	hsa-miR-154-5p	1.36	1.16E-02
hsa-miR-4751	0.82	2.96E-02	hsa-miR-654-3p	1.39	2.42E-02
hsa-miR-8089	0.82	1.15E-03	hsa-miR-6758-3p	1.40	5.15E-03
hsa-miR-7150	0.82	2.29E-03	hsa-miR-515-3p	1.40	1.87E-02
hsa-miR-7856-5p	0.82	3.95E-02	hsa-miR-5587-5p	1.43	4.12E-02
hsa-miR-6828-5p	0.83	2.36E-02	hsa-miR-3691-3p	1.43	2.12E-02
hsa-miR-3126-5p	0.83	1.78E-02	hsa-miR-2115-5p	1.43	9.40E-03
hsa-miR-8073	0.83	1.12E-03	hsa-miR-6071	1.44	6.42E-04
hsa-miR-3918	0.83	2.72E-03	hsa-miR-676-3p	1.47	3.73E-02
hsa-miR-6847-5p	0.83	4.82E-02	hsa-miR-1251-3p	1.47	4.30E-02

hsa-miR-4688	0.83	3.26E-03	hsa-miR-182-5p	1.50	2.84E-02
hsa-miR-323a-5p	0.83	3.88E-02	hsa-miR-511-3p	1.52	1.03E-02
hsa-miR-8078	0.83	2.50E-03	hsa-miR-640	1.52	2.76E-02
hsa-miR-548ah-3p	0.83	4.37E-02	hsa-miR-6864-3p	1.54	3.05E-02
hsa-miR-146a-3p	0.83	2.59E-02	hsa-miR-3680-5p	1.56	1.33E-02
hsa-miR-6876-5p	0.84	1.02E-02	hsa-miR-3615	1.56	2.86E-02
hsa-miR-1972	0.84	1.91E-02	hsa-miR-15b-3p	1.59	4.97E-02
hsa-miR-1275	0.84	1.30E-03	hsa-miR-582-5p	1.59	3.93E-02
hsa-miR-6731-5p	0.84	7.54E-03	hsa-miR-3978	1.61	4.17E-02
hsa-miR-4646-5p	0.84	3.45E-03	hsa-miR-647	1.64	1.83E-02
hsa-miR-6895-5p	0.84	8.05E-03	hsa-miR-383-5p	1.67	2.00E-02
hsa-miR-6893-5p	0.84	2.79E-03	hsa-miR-4797-3p	1.68	5.70E-03
hsa-miR-4478	0.84	2.89E-02	hsa-miR-144-5p	1.73	1.89E-02
hsa-miR-6880-5p	0.84	6.68E-03	hsa-miR-501-3p	1.74	2.71E-02
hsa-miR-3185	0.84	5.15E-03	hsa-miR-105-5p	1.74	9.79E-03
hsa-miR-7846-3p	0.85	2.23E-03	hsa-miR-499a-5p	1.95	3.63E-02

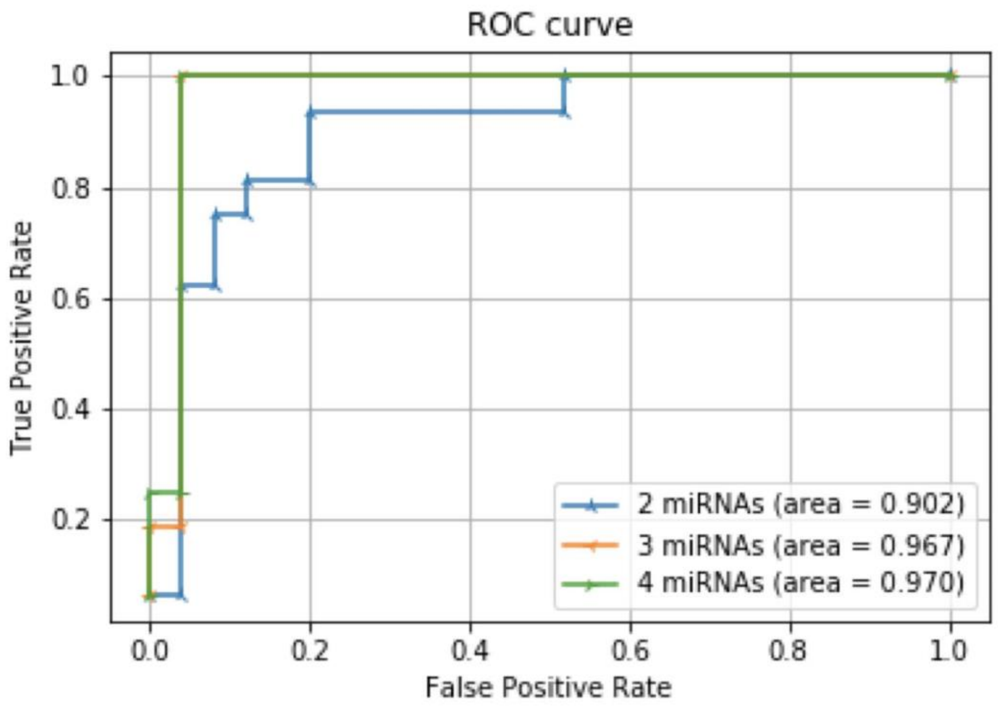
ROC curve
True Positive Rate
False Positive Rate
2 miRNAs (area = 0.902)
3 miRNAs (area = 0.967)
4 miRNAs (area = 0.970)

A

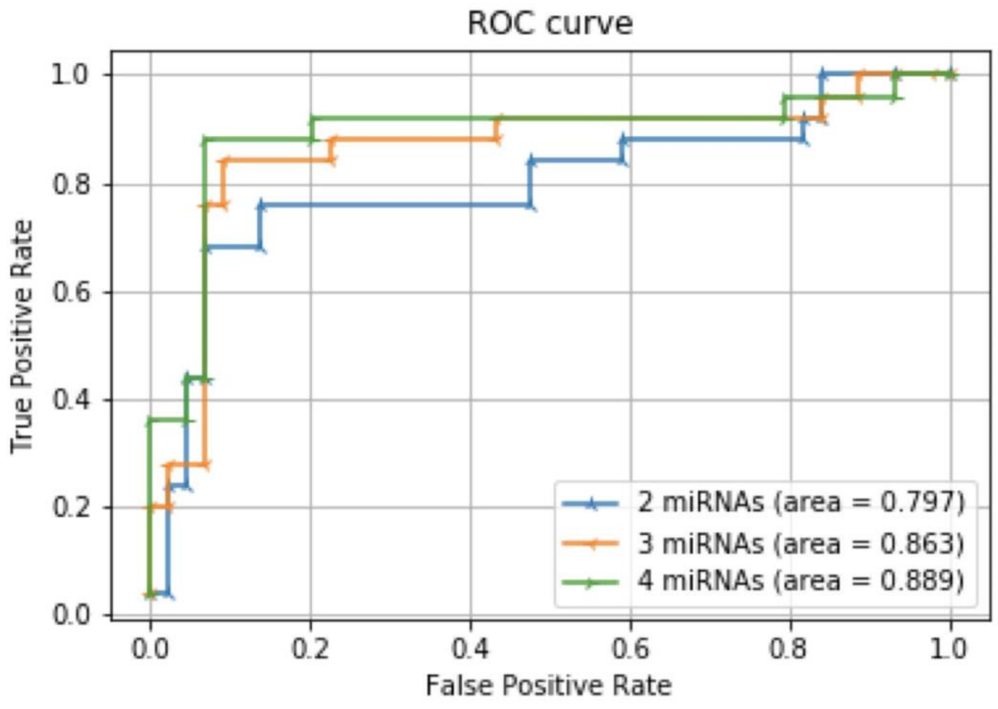
ROC curve
True Positive Rate
False Positive Rate
2 miRNAs (area = 0.797)
3 miRNAs (area = 0.863)
4 miRNAs (area = 0.889)

B

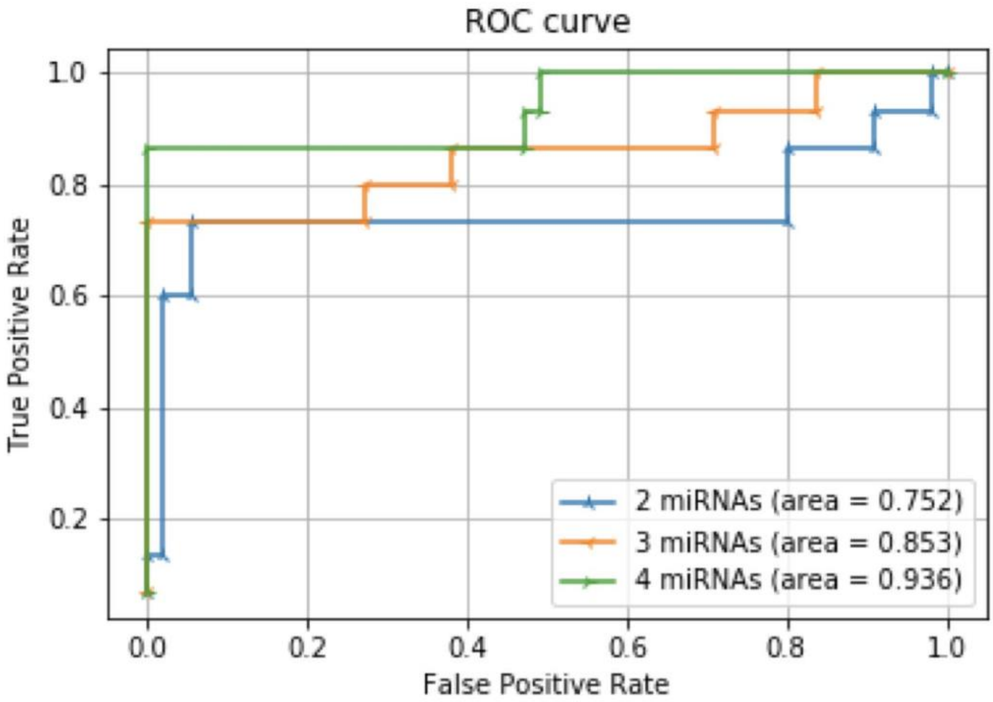

C

Figure S1. Receiver Operating Characteristic (ROC) curves for three cases: (A) Prediction of HCC recurrence in patients with liver cirrhosis using three miRNAs. (B) Prediction of HCC recurrence in non-cirrhosis and liver cirrhosis patients using four miRNAs, (C) Prediction of hepatocellular carcinoma (HCC) occurrence, where all samples were included in both the training and test datasets (because of small numbers of positive samples). In Fig. S1(A), the orange curve is largely overlapped with the green curve.

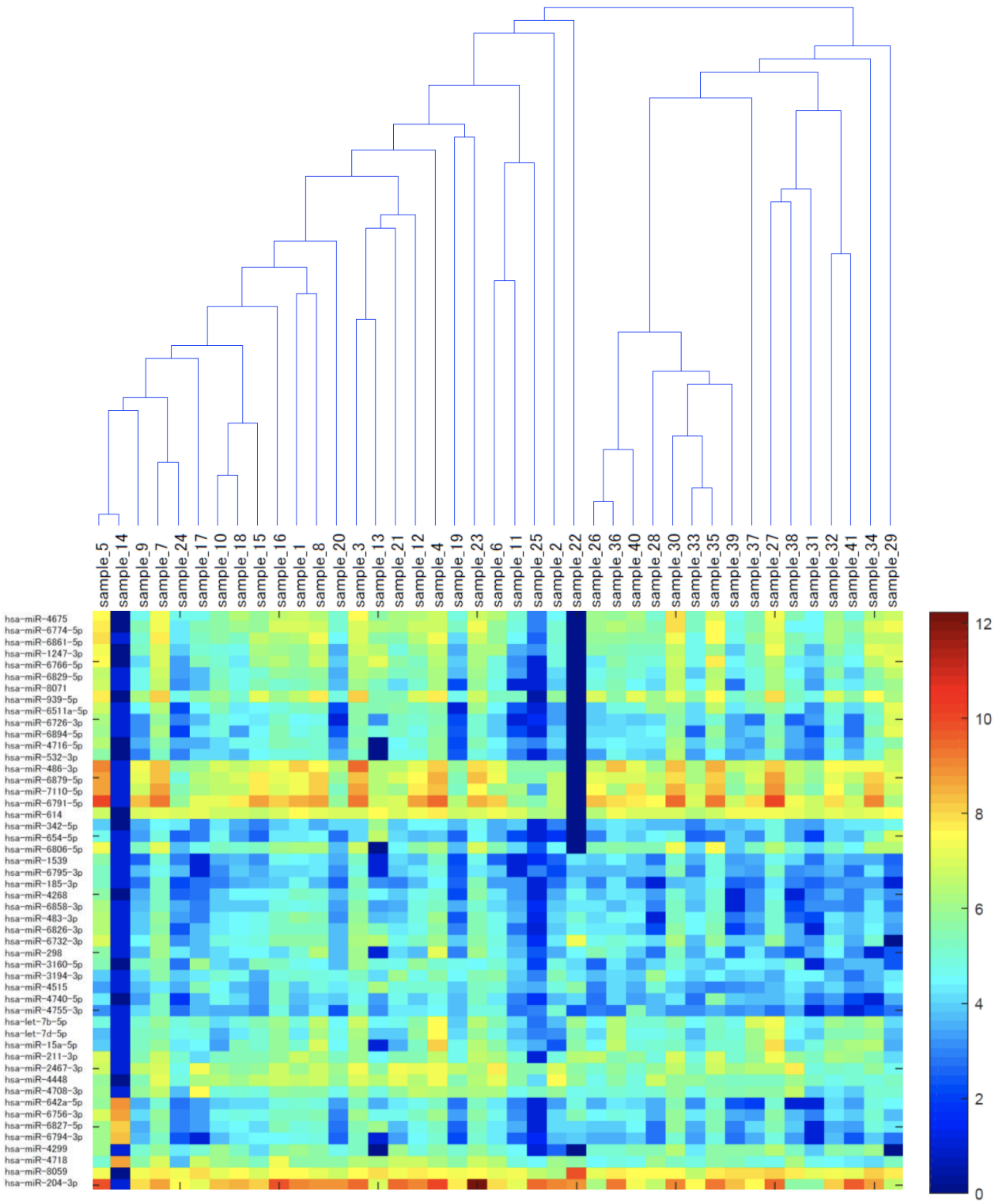
sample_5
sample_14
sample_9
sample_7
sample_24
sample_17
sample_10
sample_18
sample_15
sample_16
sample_1
sample_8
sample_20
sample_3
sample_13
sample_21
sample_12
sample_4
sample_19
sample_23
sample_6
sample_11
sample_25
sample_2
sample_22
sample_26
sample_36
sample_40
sample_28
sample_30
sample_33
sample_35
sample_39
sample_37
sample_27
sample_38
sample_31
sample_32
sample_41
sample_34
sample_29
hsa-miR-4675
hsa-miR-6774-5p
hsa-miR-6861-5p
hsa-miR-1247-3p
hsa-miR-6766-5p
hsa-miR-6829-5p
hsa-miR-8071
hsa-miR-939-5p
hsa-miR-6511a-5p
hsa-miR-6726-3p
hsa-miR-6894-5p
hsa-miR-4716-5p
hsa-miR-532-3p
hsa-miR-486-3p
hsa-miR-6879-5p
hsa-miR-7110-5p
hsa-miR-6791-5p
hsa-miR-614
hsa-miR-342-5p
hsa-miR-654-5p
hsa-miR-6806-5p
hsa-miR-1539
hsa-miR-6795-3p
hsa-miR-185-3p
hsa-miR-4268
hsa-miR-6858-3p
hsa-miR-483-3p
hsa-miR-6826-3p
hsa-miR-6732-3p
hsa-miR-298
hsa-miR-3160-5p
hsa-miR-3194-3p
hsa-miR-4515
hsa-miR-4740-5p
hsa-miR-4755-3p
hsa-let-7b-5p
hsa-let-7d-5p
hsa-miR-15a-5p
hsa-miR-211-3p
hsa-miR-2467-3p
hsa-miR-4448
hsa-miR-4708-3p
hsa-miR-642a-5p
hsa-miR-6756-3p
hsa-miR-6827-5p
hsa-miR-6794-3p
hsa-miR-4299
hsa-miR-4718
hsa-miR-8059
hsa-miR-204-3p
12
10
8
6
4
2
0

A

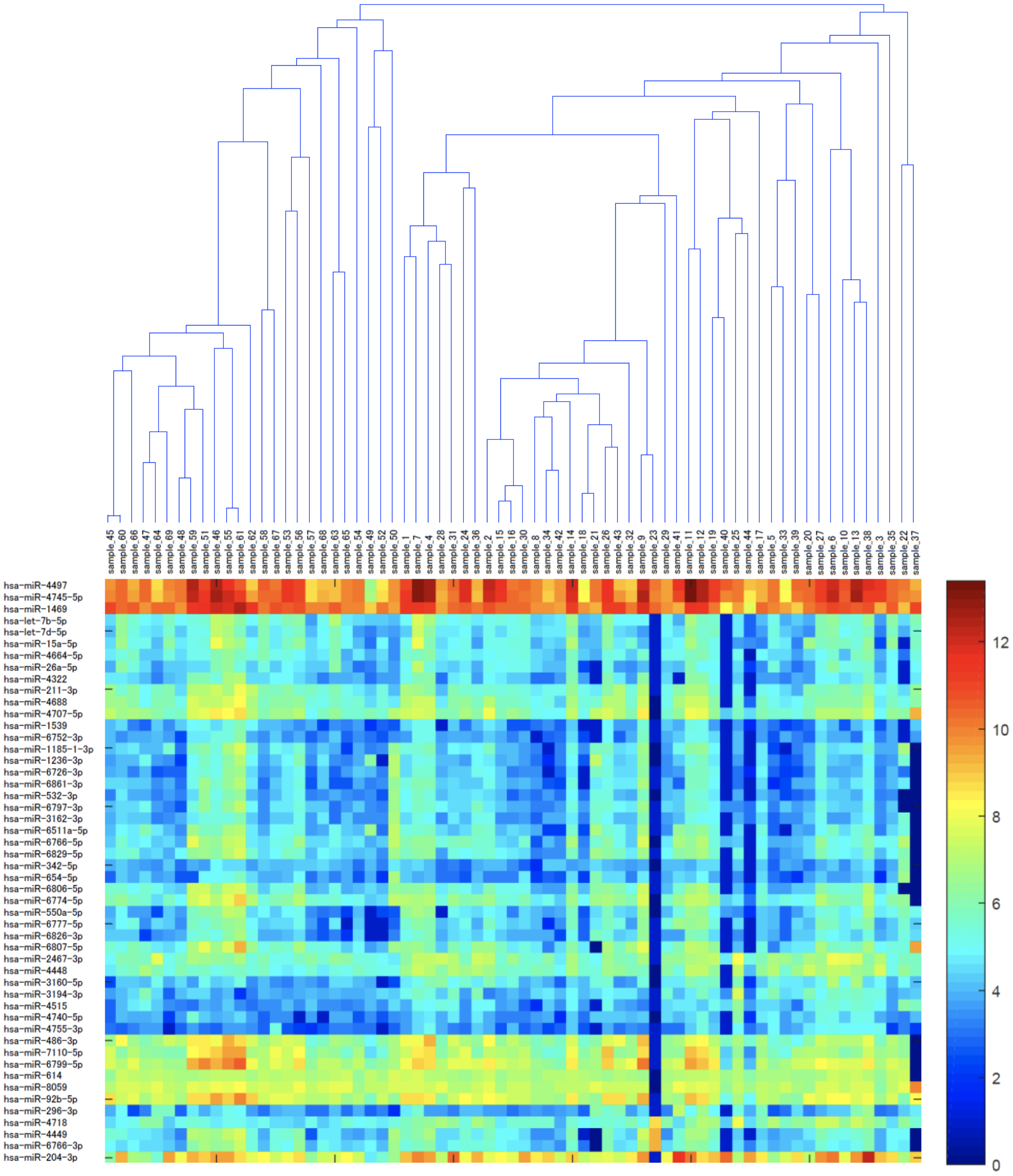
sample_45 sample_60 sample_66 sample_47 sample_64 sample_69 sample_48 sample_59 sample_51 sample_46 sample_55 sample_61 sample_62 sample_58 sample_67 sample_53 sample_56 sample_57 sample_68 sample_63 sample_65 sample_54 sample_49 sample_52 sample_50 sample_1 sample_7 sample_4 sample_28 sample_31 sample_24 sample_36 sample_2 sample_15 sample_16 sample_30 sample_8 sample_34 sample_42 sample_14 sample_18 sample_21 sample_26 sample_43 sample_32 sample_9 sample_23 sample_29 sample_41 sample_11 sample_12 sample_19 sample_40 sample_25 sample_44 sample_17 sample_5 sample_33 sample_39 sample_20 sample_27 sample_6 sample_10 sample_13 sample_38 sample_3 sample_35 sample_22 sample_37
hsa-miR-4497
hsa-miR-4745-5p
hsa-miR-1469
hsa-let-7b-5p
hsa-let-7d-5p
hsa-miR-15a-5p
hsa-miR-4664-5p
hsa-miR-26a-5p
hsa-miR-4322
hsa-miR-211-3p
hsa-miR-4688
hsa-miR-4707-5p
hsa-miR-1539
hsa-miR-6752-3p
hsa-miR-1185-1-3p
hsa-miR-1236-3p
hsa-miR-6726-3p
hsa-miR-6861-3p
hsa-miR-532-3p
hsa-miR-6797-3p
hsa-miR-3162-3p
hsa-miR-6511a-5p
hsa-miR-6766-5p
hsa-miR-6829-5p
hsa-miR-342-5p
hsa-miR-654-5p
hsa-miR-6806-5p
hsa-miR-6774-5p
hsa-miR-550a-5p
hsa-miR-6777-5p
hsa-miR-6826-3p
hsa-miR-6807-5p
hsa-miR-2467-3p
hsa-miR-4448
hsa-miR-3160-5p
hsa-miR-3194-3p
hsa-miR-4515
hsa-miR-4740-5p
hsa-miR-4755-3p
hsa-miR-486-3p
hsa-miR-7110-5p
hsa-miR-6799-5p
hsa-miR-614
hsa-miR-8059
hsa-miR-92b-5p
hsa-miR-296-3p
hsa-miR-4718
hsa-miR-4449
hsa-miR-6766-3p
hsa-miR-204-3p
12
10
8
6
4
2
0

B

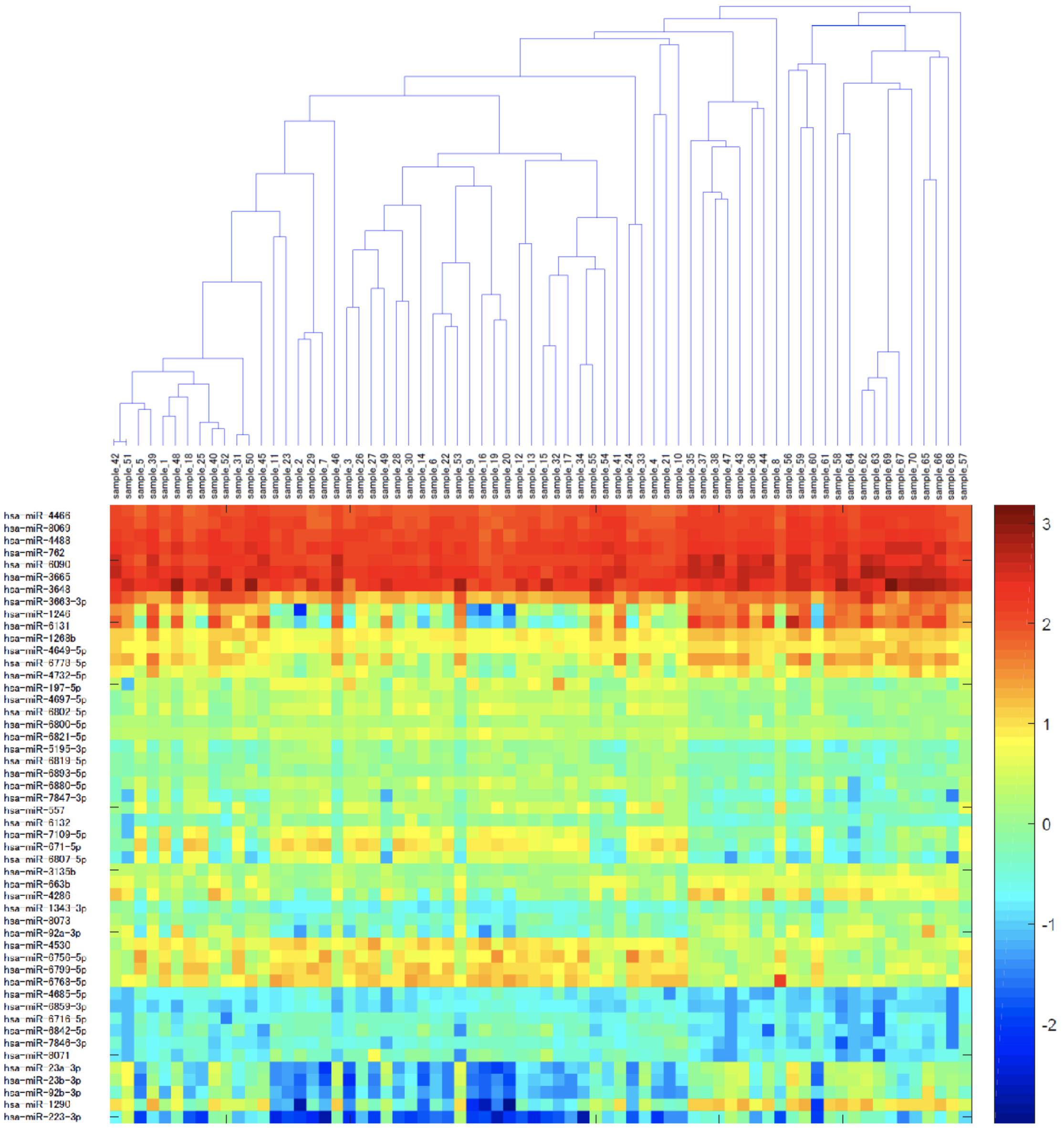

C

Figure S2. Heat maps for three cases. (**A**) HCC recurrence in patients with liver cirrhosis using three miRNAs. (**B**) HCC recurrence in non-cirrhosis and liver cirrhosis patients using four miRNAs, (C) Hepatocellular carcinoma (HCC) occurrence. Supervised clustering was applied to samples so that two classes (e.g., recurrence vs. non-recurrence) were clearly separated, where relevant 50 miRNAs selected by the procedure in Section 2.5 were used for each case. Note that samples were ordered (as a result of supervised clustering) so that positive samples (e.g., recurrent samples) were placed in the right part in (A) and (C), whereas they were placed in the left part in (B). Supervised clustering was performed using fitcsvm and linkage functions of MATLAB.
